# Lipid vesicle-based molecular robots

**DOI:** 10.1039/d3lc00860f

**Published:** 2024-01-19

**Authors:** Zugui Peng, Shoji Iwabuchi, Kayano Izumi, Sotaro Takiguchi, Misa Yamaji, Shoko Fujita, Harune Suzuki, Fumika Kambara, Genki Fukasawa, Aileen Cooney, Lorenzo Di Michele, Yuval Elani, Tomoaki Matsuura, Ryuji Kawano

**Affiliations:** a Department of Biotechnology and Life Science, Tokyo University of Agriculture and Technology 2-24-16 Naka-cho Koganei-shi Tokyo185-8588 Japan rjkawano@cc.tuat.ac.jp; b Earth-Life Science Institute, Tokyo Institute of Technology Ookayama 2-12-1 Meguro-Ku Tokyo 152-8550 Japan; c School of Life Science and Technology, Tokyo Institute of Technology Ookayama 2-12-1 Meguro-Ku Tokyo 152-8550 Japan; d Department of Chemical Engineering, Imperial College London South Kensington London SW7 2AZ UK; e Department of Chemical Engineering and Biotechnology, University of Cambridge Cambridge CB3 0AS UK; f Department of Chemistry, Molecular Sciences Research Hub, Imperial College London London W12 0BZ UK; g FabriCELL, Molecular Sciences Research Hub, Imperial College London London W12 0BZ UK

## Abstract

A molecular robot, which is a system comprised of one or more molecular machines and computers, can execute sophisticated tasks in many fields that span from nanomedicine to green nanotechnology. The core parts of molecular robots are fairly consistent from system to system and always include (i) a body to encapsulate molecular machines, (ii) sensors to capture signals, (iii) computers to make decisions, and (iv) actuators to perform tasks. This review aims to provide an overview of approaches and considerations to develop molecular robots. We first introduce the basic technologies required for constructing the core parts of molecular robots, describe the recent progress towards achieving higher functionality, and subsequently discuss the current challenges and outlook. We also highlight the applications of molecular robots in sensing biomarkers, signal communications with living cells, and conversion of energy. Although molecular robots are still in their infancy, they will unquestionably initiate massive change in biomedical and environmental technology in the not too distant future.

## Abstract 2

A molecular robot, which is a system comprised of one or more molecular machines and computers, can execute sophisticated tasks in many fields that span from nanomedicine to green nanotechnology.

## Graphical abstract

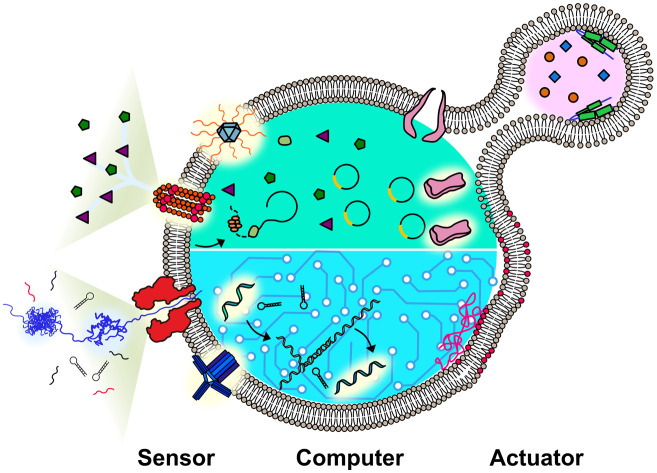

## Introduction

Machines equipped to perform specific actions have disencumbered humans of exhausting labor, helping us accomplish tasks that would be impossible with only human hands. The revolution in mechanization began in the second half of the 18th century and has led to previously unimaginable improvements in humankind's quality of life. During the past 40 years, this revolution has led to a new generation of machines with smaller sizes, pushing the boundaries of applications. In the field of organic chemistry, a breakthrough molecular catenane with two interlocked rings was reported by Jean-Pierre Sauvage in the early 1980s,^1^ subsequently inspiring other scientists to develop molecular-scale machines with complicated functions such as rotaxanes,^2^ motors,^3^ and nanocars.^4^ In 2016, three pioneers of molecular machines were awarded the Nobel Prize in Chemistry, reflecting the recognition of the broad impact of molecular machines.

The concept of molecular machines has motivated research activity in a novel field called “molecular robots”.^5^ According to the Cambridge dictionary, a robot is “a machine controlled by a computer that is used to perform jobs automatically”. By this definition, a molecular robot is a system composed of molecular-scale machines and computers that are used to execute tasks automatically. A living cell could be considered as one such miraculous robot produced by nature. With DNA serving as computers to provide solutions and proteins working as machines to perform specific functions, a living cell performs sophisticated tasks independent of human control. Taking inspiration from living cells, the ultimate goal of the field of molecular robots is to artificially construct an automated system capable of solving problems at the molecular level using molecular machines and computers.^6,7^

**Fig. 1:**
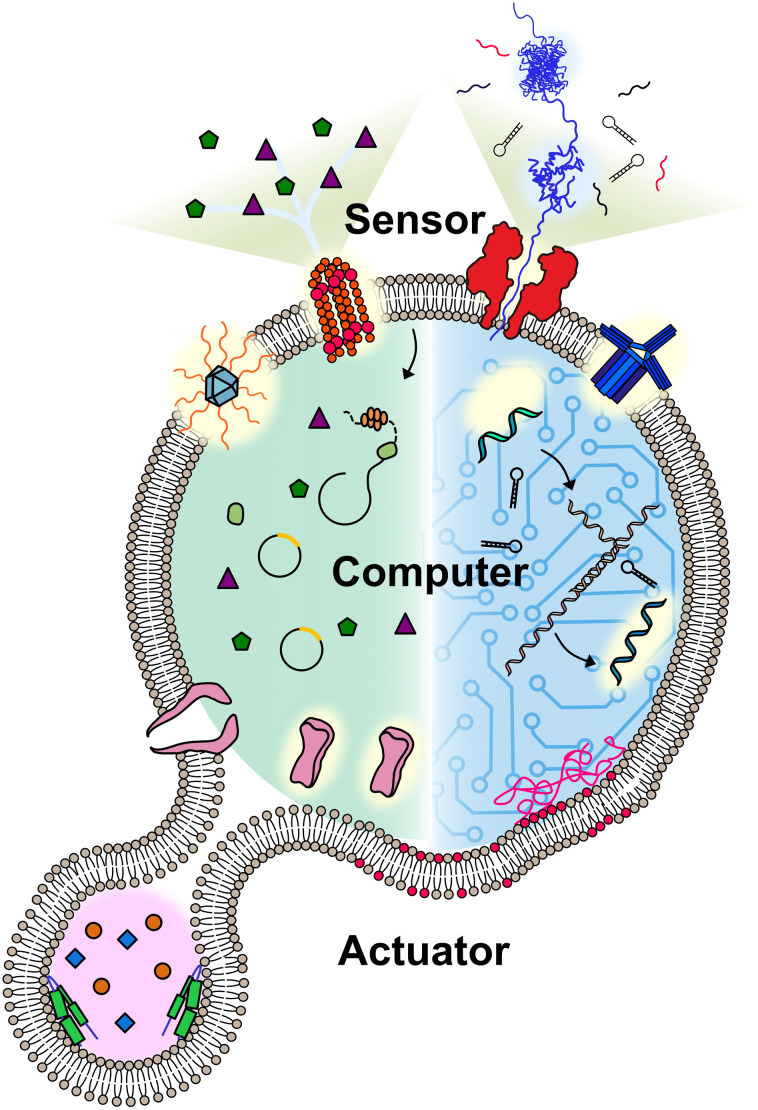
Conceptual illustration of a molecular robot including: a body to encapsulate molecular machinery, sensors to collect signals, computers to make decisions, and actuators to perform the tasks.

A molecular robot always includes some or all of the following: a body, sensors, computers, and actuators (Fig. 1). Pioneers in the field have applied hydrogels as the body of molecular robots,^5^ however, the lack of a barrier between the embeddings and environment can lead to undesired leakage. Lipid vesicles, which are comprised of lipid membranes separating an inner lumen from the outer solution, provides an alternative that could fully meet this problem. The size of the lipid vesicles can be tailored from nano- to micro-meter in diameter. For molecular robots, the micro-sized vesicles, so-called giant unilamellar vesicles (GUVs), are more desirable due to the demand for sufficient internal volume to house the sensors, computers, and actuators.^8,9^ Molecular robots require sensors in order to detect signals in the environment. This can be accomplished by ion channels or nanopores, which punch holes in lipid membranes. Nanopores can act as a signal filter, selectively transporting molecular signals based on their size or charge.^10,11^ Once the signals are transported, they can be processed and translated by molecular computing machinery. DNA computing, pioneered by L. Adleman in 1994,^12^ has evolved in recent decades into computers applicable in molecular robots, with the benefit of their capability to perform multiple parallel computations.^12,13^ An alternative choice for the computing machinery is cell-free protein synthesis (CFPS), enabling the output of proteins *in vitro* in response to the input of DNA.^14^ Actuators for molecular robots, which include DNA nanostructures,^15^ peptides,^16^ and proteins,^17^ convert signals to achieve physical movements like deformation or propulsion.

Although constant progress has been made on the underlying technology, studies into prototyping molecular robots do not have a long history. In 2014, Nishimura *et al.* incorporated CFPS into GUVs, and then, in the presence of amino acids, GFP synthesis was performed as an output.^18^ In 2017, Sato *et al.* developed GUVs equipped with actuators containing DNA clutches and microtubule motors.^15^ Once light irradiation was applied, the clutch was engaged and the shape of GUVs underwent sequential changes. These pioneering studies provide a clear path towards molecular robots with increasingly complex functions.

It should be noted that molecular robots sometimes share related technologies with the field of artificial cells. The goal of constructing artificial cells is to mimic the function of living cells, while molecular robots place great value on developing engineering applications that could help humans to perform tasks in micro or nano dimensions. There is therefore a drive to engineer molecular robots with functions exceeding those of living cells (for details on artificial cells please refer to previous reviews^19,20^). This review focuses on the recent progress and challenges in the core technologies of molecular robots and explores emerging directions. We also highlight some applications of molecular robots such as molecular sensing, communications with living cells, and energy conversion. Finally, we close with a discussion on potential research trends in this field during the coming decade.

## Body of molecular robots

Molecular robots typically require encapsulation within a compartment, which acts as a boundary, separating the interior from the exterior environment. Various types of compartments have been employed to date, including lipid membranes,^21^ hydrogels,^22^ block co-polymers,^23^ DNA droplets,^24^ and coacervates,^25^ each offering distinct advantages and limitations. Some researchers have explored the formation of hybrid chassis by combining different compartment types, leveraging the advantages associated with each constituent part. For instance, coacervate or DNA/hydrogel systems interfaced with lipid membranes can be combined to enhance functionality.^26–28^

Among these compartment types, lipid vesicles are the most commonly used for several reasons. Firstly, they are biomimetic, closely resembling biological membranes from a chemical and morphological perspective. This characteristic enables facile incorporation of membrane-bound molecular machinery, including membrane proteins, nanopores, and receptors, thereby imparting specific functionalities into the membranes.^29,30^ For example, the controlled flux of cargo molecules in response to stimuli,^30,31^ which can be used to mediate responses in living cells.^32^

Furthermore, lipid vesicles are chemically inert and highly efficient at compartmentalizing large charged molecules from the surrounding environment, creating a chemically distinct internal environment. This feature enables researchers to exploit the diversity of lipid building blocks, both synthetic and biological, to create functional membranes with diverse behaviors. Examples include membranes capable of self-assembling into tissue-like structures,^33^ membranes that can disassemble and reassemble in response to physicochemical cues to reshuffle material between them,^34^ and membranes that release cargo triggered by light, temperature fluctuations, magnetic fields, or biomarkers.^31^ Such versatility opens up exciting possibilities for molecular robotics and targeted drug delivery systems, among other applications.

**Fig. 2:**
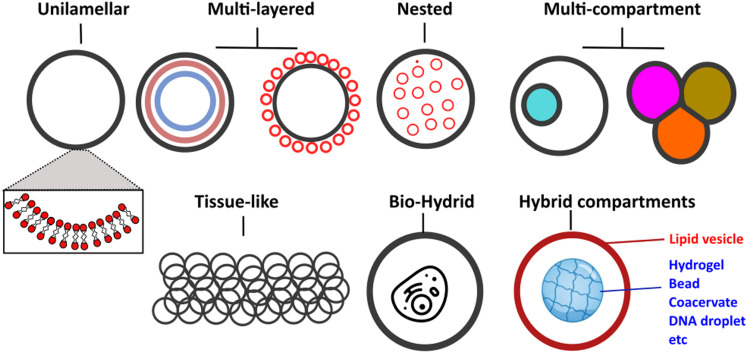
Schematic showing examples of different vesicle architectures that can be controllably manufactured using microfluidic techniques.

Vesicles can be classified primarily based on their size and lamellarity.^35^ GUVs have a diameter of approximately 2 μm and above (making them cell-sized vesicles), comprising a single lipid membrane (as opposed to multi-layered onion-like structures known as multilamellar vesicles). Other types of vesicles include small and large unilamellar vesicles, which fall into the sub-micron size range, as well as multi-vesicular vesicles (multisomes). Additionally, there have been intriguing examples of hybrid structures, where vesicles of different types are assembled into more architecturally complex arrangements, such as nested^36^ or layered geometries.^37^ A schematic of the different architectures it is now possible to generate microfluidic techniques and principles in biomembrane engineering is shown in Fig. 2.

In molecular robotics, GUVs are often the go-to architecture due to their resolvability by optical microscopy. Moreover, they allow for efficient encapsulation of large and highly charged building blocks (DNA, proteins, nanoparticles, and even whole organelles/cells),^38,39^ which becomes more problematic when using vesicles in the sub-micron regime. For instance, to encapsulate the biomolecular components required for transcription and translation, precise stoichiometries of plasmids, ribosomes, polymerases, and around 40 other molecular components must be co-encapsulated.^40^ Achieving such precision becomes statistically improbable in lower-size regimes. GUVs thus provide a more suitable environment for accommodating these complex biomolecular systems.

### Vesicle generation strategies

To generate GUVs, various methods are available, with several comprehensive reviews published in this area.^41–43^ When evaluating the different methods, it is essential to consider the relevant Key Performance Indicators (KPIs), which include:

#### (i) Encapsulation efficiency

The method's efficacy in encapsulating molecules may vary based on the type of molecule involved. In molecular robotics applications, achieving a high encapsulation efficiency for large, charged species (such as DNA, proteins, nanoparticles, *etc.*) is crucial, as they contribute to the system's functionality.

#### (ii) Monodispersity and control over size

The ability to produce vesicles with well-defined dimensions is of paramount importance, as size directly influences downstream applications, and consistent sizes ensure consistent performance.

#### (iii) Control of architecture and lipid content

Ideally, the method should offer precise control over the lipid composition of the vesicle membrane, ensuring uniformity, and yielding unilamellar membranes (or membranes of a defined multi-lamellarity).^44,45^

#### (iv) Generation throughput

For practical applications, it is advantageous to produce significant quantities of vesicles within a reasonable time frame to meet industrial demands efficiently.

#### (v) Presence of impurities

Some applications, particularly those related to fundamental biological studies, benefit from vesicle membranes free of any impurities and oil.

Considering these KPIs when assessing the different GUV generation methods will aid researchers in selecting the most appropriate technique for their specific applications, ensuring optimal performance and reliable outcomes.

Classical methods of generating GUVs include gentle hydration, gel-assisted swelling, and electroformation.^35^ These techniques have played a pivotal role in revolutionizing our understanding of membrane biophysics phenomena. They provided crucial insights into the mechanical properties of membranes, the influence of lipid composition, the coupling between mechanics and membrane protein activity, as well as the principles governing phase behavior and coexistence of domains (lipid rafts) in cell membranes.^46^ However, for molecular robotics applications, these methods have proven largely unsuitable due to their limitations. They suffer from poor encapsulation efficiency, uncontrolled production, and yield polydisperse populations concerning size, architecture, cargo, and lipid composition.

**Fig. 3:**
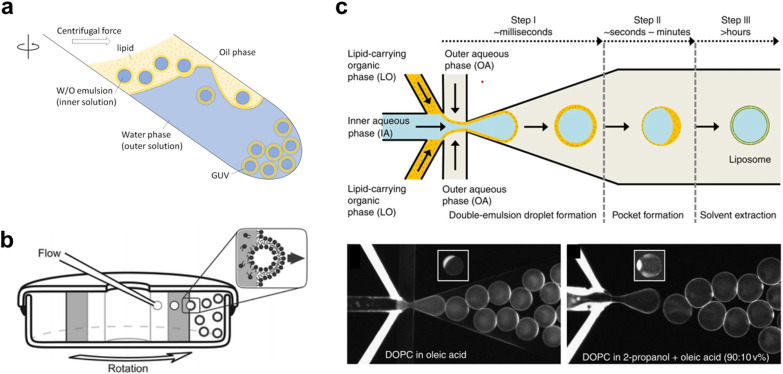
Examples of different techniques used to generate Giant Unilamellar Vesicles (GUVs). a, Emulsion phase transfer, where lipid stabilised w/o droplets are taken through a w/o column through a centrifugal force, resulting in the formation of a second monolayer, and hence the establishment of a lipid bilayer vesicle.^354^ b, Continuous droplet interface crossing encapsulation (cDICE) involving instrumentation which houses concentric layers of fluids with different densities, where aqueous droplets that emerge from a capillary are driven by centrifugal force through multiple lipid interfaces.^355^ c, Octanol-assisted liposome assembly (OLA) method of generating lipid vesicles on-chip. This method involves generating a lipid stabilised w/o/w double emulsion followed by extraction of the intermediate oil phase, leaving behind a lipid membrane.^356^ Figures reproduced with permission from: a, ref. 354, copyright 2023, MDPI; b, ref. 355, copyright 2016, the Royal Society of Chemistry; c, ref. 356, copyright 2016, Nature Publishing Group.

In the past two decades, innovations have led to the emergence of a new class of vesicle fabrication strategies based on water/oil emulsion technology^41,47^ (Fig. 3(a)). These methods rely on the formation of water/oil droplets stabilized by lipid monolayers. When a second lipid-stabilized water/oil interface is deposited on top of these droplets (*i.e.*, when they are transferred from a bulk oil phase to a water phase), it templates the formation of a second monolayer, resulting in the formation of a lipid membrane. These techniques are sometimes referred to as emulsion transfer or phase transfer methods.

The simplest version of this method involves the manual formation of a water/oil emulsion through pipetting and/or agitation, followed by phase transfer under gravity, often with the aid of a centrifuge. This process can be repeated multiple times to yield multi-layered arrangements.^45^ This method is highly adaptable for translation into a microfluidic format, where devices can be created using soft-lithography,^48,49^ glass capillaries,^50^ or 3D printing.^51^ On-chip droplet production and transfer across a water/oil interface can be achieved using microfabricated pillars^52^ or flow-based approaches.^53^ Another approach involves using bespoke instrumentation to enable the passage of monodisperse droplets through a continuously rotating horizontal column at the water/oil interface, a method known as cDICE (continuous droplet interface crossing encapsulation), depicted in^54^Fig. 3(b).

An alternative technique employs microfluidics to form a lipid-stabilized water/oil/water double emulsion. Upon evaporation or de-wetting of the intermediate oil phase, this process leads to the formation of GUVs.^49,55^ The most commonly used version of this method employs octanol to promote de-wetting and is therefore referred to as octanol-assisted liposome assembly (OLA) (Fig. 3(c)).

### Advantages of microfluidics

The use of microfluidics enables high-throughput manufacturing of monodisperse (<5% C.V.) GUVs with defined sizes (*ca.* 5–200 μm diameter) and remarkably high encapsulation efficiency (approaching 100%).^41^ In the context of creating, manipulating, and analysing molecular robotic systems based on vesicle structures, other lab-on-chip microfluidic modules come into play. For instance, microstructure traps have been employed to capture arrays of hundreds of vesicles, facilitating high-throughput experimentation and extended analysis.^56^ These traps have been coupled with valves to control the perfusion of materials for permeation studies. Purification modules have also been included to remove unencapsulated material and debris from the surroundings.^48^

Different chip geometries can be utilized to generate multi-compartment architectures, such as vesicles-in-vesicles and arrangements with compartments side-by-side.^50,57^ By functionalizing compartments with enzymes, DNA programs, nanoparticles, and CFPS, various features like signalling cascades, trans-compartment communication, division, and spatial segregation of reactions have been achieved within these microrobots.^19,20^ Moreover, linking up thousands of vesicles through adhesive membrane patches has led to the creation of tissue-like structures with self-healing properties.^33^ Related technologies include opto-fluidic methods that employ optical trapping and laser technologies for the on-demand fusion of compartments. These techniques trigger biochemical reactions in femtoliter (pL/fL) reaction vessels, study architectural transformations induced by lipid mixing,^58^ and manipulate raft-like domains on the vesicle surface.^59^

## Sensors of molecular robots

In living cells, membrane receptors, transporters, and ion channels work as sensors to help cells respond to chemical and physical stimuli. Such functionalities have also been exploited to implement sensing capabilities in molecular robots. In particular, nanopores forming stable nanoscale openings across lipid membranes have been shown to mediate transport of large molecules, enabling their detection by the molecular robots.^7^ The opening and closing of nanopores (gating) can be regulated by environmental stimuli (*e.g.*, pH, light, temperature, osmotic pressure), further enhancing the sensing capabilities of the molecular robots. Various materials have been utilized to assemble nanopores including proteins,^60^ peptides,^61^ DNA,^11^ and synthetic materials.^62^ In the current section, we will mainly focus on the characteristics, differences, and recent progress of nanopore assembly using different building materials, and we will also describe some unique approaches that introduce membrane receptors to GUVs. (For more details on the introduction of membrane receptors to GUVs, we direct readers to a review by Tosaka *et al.*^63^).

### Protein nanopores

**Fig. 4:**
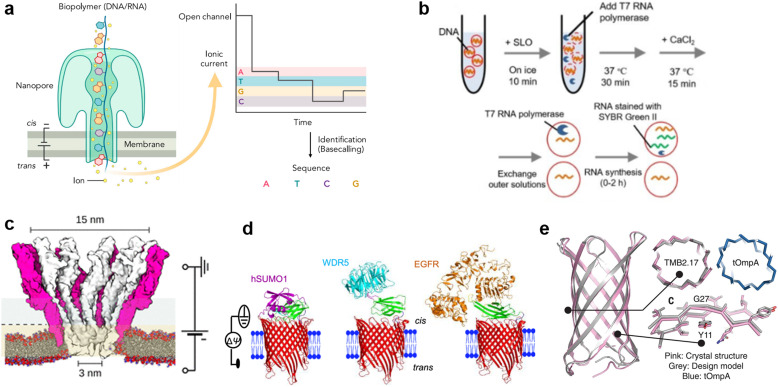
Protein nanopores for transmitting information. a, Illustration of the mechanism of nanopore DNA sequencing.^75^ b, Transport of T7 RNA polymerase by the SLO nanopore in a lipid vesicle system.^85^ c, Molecular surface illustration of the YaxAB nanopore.^86^ d, Protein detection with a monobody containing tFhuA nanopores.^94^ e, A comparison of the crystal structure (pink) and the designed structure (gray) of a *de novo*-designed transmembrane eight-strand β-barrel.^99^ Figures reproduced with permission from: a, ref. 75, copyright 2021, Elsevier; b, ref. 85, copyright 2021, Wiley-VCH; c, ref. 86, copyright 2023, American Chemical Society; d, ref. 94, copyright 2023, Nature Publishing Group; e, ref. 99, copyright 2021, AAAS.

Protein nanopores are the most widely-used type of nanopore, both in planar lipid membrane systems and lipid vesicle systems. When reconstituted in planar membrane systems, nanopores are powerful tools for single molecule detection. The target molecules are detected by recording the change in ionic current output as the molecules pass through nanopores under a constant voltage input. Since the detection sensitivity of nanopore sensing largely depends on the size and structure of the pores, a wide range of proteins have been investigated for the detection of specific targets.^60,64^ In 1996, α-hemolysin (αHL), with a pore size of 1.4 nm at the restriction, which is compatible with the translocation of single-stranded DNA (ssDNA), was initially employed by Kasianowicz *et al.* for detecting polynucleotides.^65^ In 2010, *Mycobacterium smegmatis* porin A (MspA) was found to be suitable for discriminating individual nucleotide bases.^66^ The above pioneering works finally led to the release of the first commercial nanopore DNA sequencer for general use in 2015 by Oxford Nanopore Technologies, using the *E. coli* curli transport channel, CsgG.^67^ Following the achievement of nanopore DNA sequencing (Fig. 4(a)), single-molecule analysis of folded proteins^68–72^ and amino acid sequences in proteins^73–75^ are now underway. Advances such as the detection of single amino acid mutations in peptides using aerolysin from *Aeromonas hydrophila*,^76^ sequential readout of peptides using MspA,^77,78^ and identification of digested protein fragments using fragaceatoxin C from *Actinia fragacea* (FraC)^79^ have been reported in recent years.

Toward the realization of molecular robots, enormous efforts have been made to construct a protein nanopore-lipid vesicle system. Similar to planar lipid membrane systems, the transport of molecules in the lipid vesicle system is highly dependent on the size of the protein nanopores. In addition to conventional studies using protein nanopores of different sizes to transport fluorophores, there are reports on the use of protein nanopores to transport molecules into lipid vesicles, including: the transmission of pH information by a voltage-dependent anion channel (VDAC, 2.5–3 nm) from yeast mitochondria,^80^ the light-/pH-dependent release of fluorescent molecules by a mechanosensitive channel of large conductance (MscL, 3 nm) from *E. coli*,^81,82^ the transport of ATP^83^ and UTP^84^ by αHL (1.4 nm) from *Staphylococcus aureus*, and the translocation of T7 RNA polymerase (98 kDa) by streptolysin O (SLO, 25–30 nm) from group A, C, and G streptococci^85^ (Fig. 4(b)).

Although numerous protein nanopores with pore diameters ranging from 1.2 to 42 nm have been reported,^60^ the investigation of novel protein nanopores remains intriguing due to the need to expand the toolbox for new target molecules. In 2023, the Maglia group reported the detection of proteins of different sizes using an α-helical pore-forming toxin derived from *Yersinia enterocolitica*, YaxAB.^86^ It consists of a dimer of YaxA and YaxB, forming assemblies of varying sizes, ranging from 8 to 12 dimeric subunits. The characteristic feature of this pore is its huge conical shape with 15/3.5 nm *cis*/*trans* openings (Fig. 4(c)). Notably, it is the largest protein nanopore ever used for the analysis of folded proteins in planar membrane systems, with the ability to capture a wide range of proteins (from 35–125 kDa). YaxAB exhibits a greater electroosmotic flow under voltage-applied conditions compared to existing nanopores, which aids the entrapment of proteins within the nanopore. Using YaxAB, the group successfully discriminated C-reactive protein (CRP, 125 kDa), hemoglobin (HG, 64 kDa), streptavidin (SA, 53 kDa), and bovine thrombin (BT, 35 kDa) from their mixture. Moreover, CRP was detected at clinically relevant concentrations (>2.5 mg L^−1^) in 160-fold diluted depleted human serum.

It is also challenging to detect molecules that are larger than the pore, or to selectively discriminate molecules of a similar size and hydrodynamic radius. Binding of target-specific moieties such as partial antibody domains,^87^ aptamers (single-stranded nucleic acids with the ability to bind to specific molecules),^88,89^ biotin (for the avidin-biotin complex),^90^ inhibitory peptides,^91^ protein receptors,^92^ and gold nanoclusters,^93^ have been employed to achieve the detection of large molecules,^87,88,90,91^ or selective discrimination.^89,92,93^ Since the configuration of the detection system changes depending on the recognition element used, it has been necessary to engineer a distinct system for each target protein. Recently, Movileanu and colleagues reported a general system for the sensitive detection of target proteins.^94^ In their study, the tFhuA nanopore, a β-barrel scaffold of ferric hydroxamate uptake component A (FhuA) from *E. coli*, is covalently attached to a monobody, a recombinant protein based on the fibronectin type III (FN3) domain (Fig. 4(d)). The advantages of monobodies are that they can be selected from a broad range of variants against numerous target proteins, and their relatively small hydrodynamic radius that does not interfere with pore opening, unlike the larger antibody-derived proteins. Using the monobody-conjugated tFhuA, the authors detected human small ubiquitin-related modifier 1 (hSUMO1), WD40 repeat protein5 (WDR5). Moreover, the group achieved the identification of the 20 nM-epidermal growth factor receptor (EGFR), which is a 180 kDa prognostic protein biomarker associated with lung, colon, and breast cancer, in 5% (v/v) fetal bovine serum (FBS), suggesting the potential applications of molecular robots in nanomedicine.

In recent years, in addition to using natural membrane proteins, attempts have been made to fabricate nanopores through *de novo* design. This approach uses computer-aided protein design to create proteins with tailored shapes and functionalities.^95–98^*De novo* design allows the fabrication of nanopores with arbitrary structures that do not exist in nature, thereby expanding the range of detectable molecules. Conventionally, *de novo* design of transmembrane proteins is considered difficult due to the unclear relationship between membrane insertion, folding mechanisms and the corresponding amino acid sequences. In 2021, Vorobieva *et al.* achieved the first *de novo* design of an eight-strand transmembrane β-barrel^99^ through a complex optimization process including: (i) the refinement of the loop structure and adjustment of hydrophobicity, (ii) backbone design and sequence optimization using Rosetta,^100^ (iii) additional design guidelines appropriate for the membrane environment, and (iv) exhaustive expression trials. The expressed structure was confirmed to be highly consistent with the designed structure (1.1 Å backbone RMSD over all residues) (Fig. 4(e)). Single-molecule detection by this protein nanopore has not yet been reported, probably due to the smaller pore size compared to natural nanopores. However, with the recent popularization of *de novo* protein design tools,^100–102^ the development of protein nanopores suitable for molecular robotics applications with better molecular selectivity, transport capacity, stability, and durability are expected in the future.

### Peptide nanopores

**Fig. 5:**
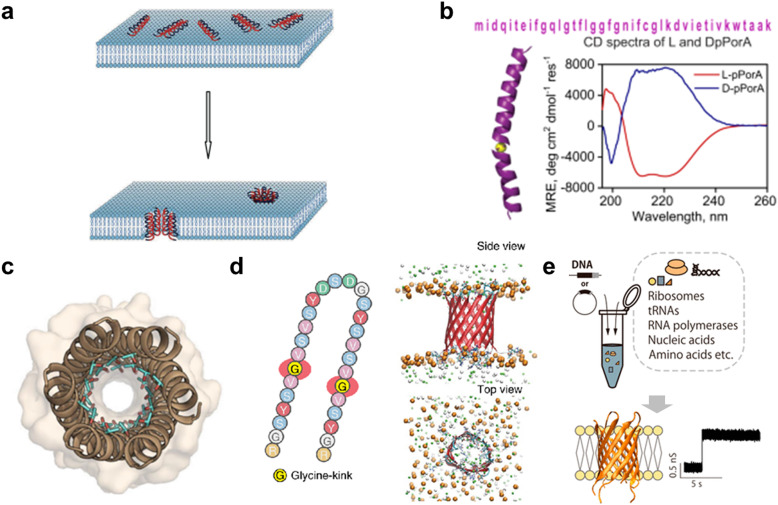
Peptide nanopores for transmitting information. a, Formation of nanopores in lipid membranes by alamethicin.^357^ b, DpPorA peptide sequence and CD spectra for DpPorA (blue) and LpPorA (red).^118^ c, X-ray crystal structure of the nanopore formed by the *de novo*-designed α-helix peptide.^358^ d, SVG28 sequence and simulated pore structure.^122^ e, Utilizing CFPS to synthesize variants of SVG28.^123^ Figures reproduced with permission from: a, ref. 357, copyright 2014, Hindawi Publishing Corporation; b, ref. 118, copyright 2022, Nature Publishing Group; c, ref. 358, copyright 2022, American Chemical Society; d, ref. 122, copyright 2023, Nature Publishing Group; e, ref. 123, copyright 2023, American Chemical Society.

As with protein nanopores, peptides that assemble into pores in the membrane are also attractive as sensors in molecular robotics. Compared with protein nanopores, peptide nanopores are more tractable, since stable peptide nanopores can be designed based on naturally occurring sequences or by *de novo* design, and peptides with the 30–40 residues required for membrane-spanning can be synthesized in large quantities by chemical synthesis. Originally, antibacterial peptides (AMPs) such as gramicidin and alamethicin were known to assemble to form pores in bacterial membranes, exhibiting antibacterial activity^61,103–106^ (Fig. 5(a)). The AMP nanopores have been reported to enable permeation of small molecules such as fluorophores,^107–111^ but it has proven difficult to transport molecules with larger molecular weights.^112–114^ In recent years, attempts have been made to design more stable and monodispersely sized peptide nanopores compared to AMP nanopores.

One approach to obtaining stable peptide nanopores is the redesign of the transmembrane regions of natural membrane proteins.^115,116^ The advantage of this approach is that no explicit complex design principles are required, and a certain degree of stability is expected. Redesigned peptide nanopores have demonstrated that such transmembrane peptides stably form pores with a certain number of monomers, opening the field of peptide nanopores and providing promising nanopore-forming peptides to date. Recently, the Mahendran group reported that DpPorA, which is a d-amino acid-substituted pPorA peptide derived from the porin PorACj of *Corynebacterium jeikeium*, could form a stable monodisperse octameric pore^116–118^ (Fig. 5(b)). Compared to conventional helix barrels, DpPorA is characterized by very large conductance and is highly cation-selective, so that cyclodextrins and nonaarginine (R9) can translocate under applied voltage in a planar membrane system, while there is negligible interaction with anionic cyclodextrins and oligopeptides. Notably, whilst their L counterparts are readily degraded by proteinase K, DpPorA is highly resistant to protease K treatment, indicating promising potential for use as a stable sensor in molecular robots in biological environments.

Similar to protein nanopores, another approach to obtain peptide nanopores is *via de novo* peptide design. Several *de novo*-designed α-helix peptides have been reported in recent years,^119,120^ as α-helices allow for the evaluation of inter-helix stability and the precise control of packing interfaces. In 2021, Woolfson and colleagues reported the *de novo* design of an α-helix peptide that forms stable, monodispersely sized pores.^120^ They began by designing a water-soluble α-helical barrel by introducing polar residues on the lumen of the α-helical bundles of pentameric or higher order (Fig. 5(c)), to support the helical interface and the passage of water molecules, resulting in a parallel hexamer water-soluble pore. By incorporating hydrophobic leucine and membrane interface-preferring tryptophan residues into the outer surface of the water-soluble pore, peptides that form monodisperse pores in the membrane were achieved. To further improve the inter-helix packing, computational scoring was used to fabricate possibly hexameric nanopores with a longer lifetime and fewer open–close states (gating). Although single-molecule detection has not been attempted, probably due to the small pore size, the group has demonstrated the fabrication of water-soluble helical barrels from 5 to 9-mers by changing the residue size at specific positions on the helical wheel,^121^ leading to the possibility of pore size expansion and greater molecular transport ability.

In 2022, Shimizu *et al.* reported the *de novo* design of β-hairpin peptides SV28 and SVG28, which form transmembrane β-barrel nanopores following four simple design principles: (i) alternating hydrophilic and hydrophobic transmembrane residues, (ii) introduction of membrane interface-anchoring residues, (iii) introduction of charged residues for membrane insertion by electrophoresis, and (iv) introduction of a glycine kink to reduce the strain of the β-barrel (only for SVG28)^122^ (Fig. 5(d)). SVG28 forms almost monodisperse heptameric pores under optimized conditions and can discriminate between L-PLL (molecular weight 30 000–70 000) and S-PLL (molecular weight 10 000). In addition, SVG28 detects cationic peptides more efficiently than the commonly used αHL nanopore due to its terminal charge. Moreover, Fujita *et al.* have found that a hydrophilic variant of SVG28 can be synthesized using CFPS, which retained the pore-forming ability^123^ (Fig. 5(e)), promising easy access to peptide nanopores for molecular robotics. Furthermore, we showed that SV28, an antecedent of SVG28, can form a wide range of pore sizes from 1.7 to 6.3 nm and can translocate the G4 structure of DNA when the pore size is sufficiently large. Therefore, we believe that it is possible to find SVG28 variant sequences that form nanopores with a suitable size for molecular transport. Future efforts are required not only to design assembled pore structures but also to add vestibule regions^124^ and lining structures^125^ that stabilize the pore structure.

### DNA nanopores and receptors

**Fig. 6:**
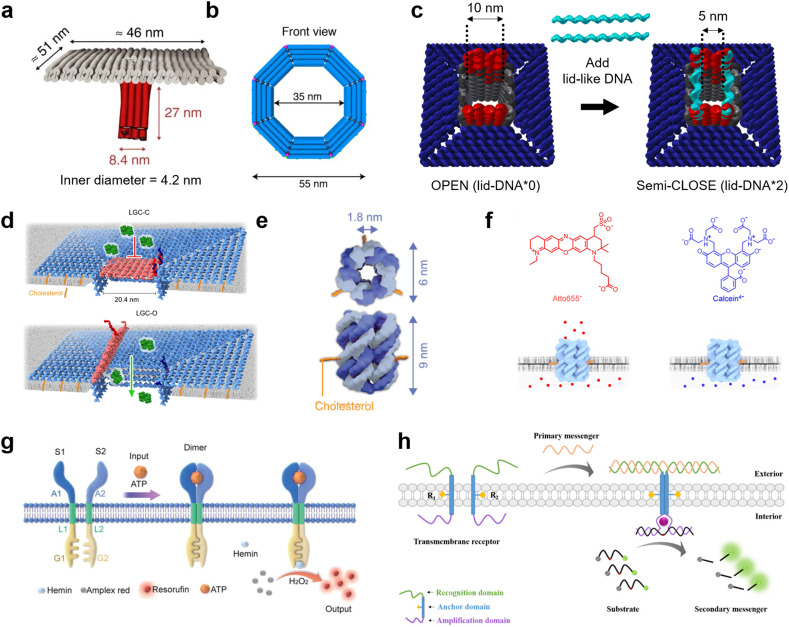
DNA nanopores and receptors for transmitting information. a, First reported DNA origami nanopore that transported dye molecules across the lipid membranes of GUVs.^132^ b, DNA origami nanopore with the largest inner diameter.^135^ c, DNA origami nanopore for size-selective transport, controlled by changing the number of lid-like DNA strands on top of the pore.^134^ d, DNA origami nanopore that reversibly opens and closes upon addition of key DNA strands.^137^ e, DNA nanopore composed of six DNA strands.^359^ f, Charge selective transport of dye molecules by a six-helix DNA nanopore.^139^ g, Receptor-mimicking DNA nanostructures capable of detecting ATP and lysozyme.^149^ h, Receptor-mimicking DNA nanostructures capable of detecting DNA strands.^150^ Figures reproduced with permission from: a, ref. 132, copyright 2016, Nature Publishing Group; b, ref. 135, copyright 2021, American Chemical Society; c, ref. 134, copyright 2021, Royal Chemical Society; d, ref. 137, copyright 2023, Nature Publishing Group; e, ref. 359, copyright 2023, Wiley-VCH; f, ref. 139, copyright 2021, American Chemical Society; g, ref. 149, copyright 2021, Royal Chemical Society; h, ref. 150, copyright 2021, American Chemical Society.

DNA nanotechnology presents a unique tool for mimicking the function of biological nanopores as sensors for molecular robots, due to its advantages in design flexibility and programmability. Nanopores formed by DNA nanotechnology, so-called DNA nanopores, can be realized by DNA origami strategies which use a long single-stranded scaffold DNA and hundreds of staple DNA strands,^126,127^ as well as DNA nanostructures strategies which use few short DNA strands.^128,129^ To embed the DNA nanopores into lipid membranes, the DNA nanopores carry hydrophobic molecules such as cholesterol and tocopherol so that they can interact with the hydrophobic membrane core. From the first two groundbreaking DNA nanopores that transported ions through planar lipid membranes,^130,131^ in 2016, Krishnan *et al.* then reported the successful transport of small dye molecules through lipid membranes of GUVs using square-shaped DNA nanopores with a 4.2 nm-wide channel lumen^132^ (Fig. 6(a)). Since then, several DNA nanopores that function on GUV membranes have been reported.^133–136^

The current strategies to improve DNA nanopores constructed by DNA origami mainly focus on two areas: expanding the pore diameter,^133–137^ and adding unique functions for selective transportation.^133,134,137^ A larger pore not only allows larger molecules to be transported, but also improves the efficiency of transport. In 2021, a 35 nm-wide DNA nanopore was reported with the capability of transporting dextran with a molecular weight of up to 250 kDa^135^ (Fig. 6(b)). Furthermore, in 2022, Xing *et al.* developed DNA nanopores with different polygonal shapes and sizes that permit the transport of antibodies.^138^ Meanwhile, adding functions such as control over gating can provide the ability to selectively transport molecules. To date, various methods for controlling the gating have been demonstrated, including: modifying the pore lumen with PEG molecules,^133^ changing the number of lid-like DNA strands on top of the pore^134^ (Fig. 6(c)), and reversible opening and closing with key DNA strands^137^ (Fig. 6(d)).

While DNA origami is advantageous in constructing pores with large diameters, their versatility and stability is hindered by the complicated design procedure. In contrast, DNA nanopores based on DNA nanostructures have generated great interest due to their simpler design strategy. To date, DNA-nanostructure-based nanopores with six-helix^11,139–141^ (Fig. 6(e)) or eight-helix^142^ bundle structures, and wireframes of triangular^143^ and square^144^ shapes have been reported to function on GUV membranes. The relatively small lumen size provides the merit of charge-selective molecular transport, since negatively-charged DNA molecules have strong electrostatic interactions with charged cargo molecules^11,139^ (Fig. 6(f)). Moreover, similar approaches have been described to control the gating of DNA nanopores with inputs including specific molecules,^145^ temperature,^146^ light,^147^ and mechanical stimuli.^148^

Receptor-mimicking DNA nanostructures, which do not puncture a pore but simply span the lipid membrane, are also potential sensors for molecular robots. Unlike DNA nanopores, receptor-mimicking DNA nanostructures do not suffer from leakage issues, and hence can achieve signal transduction with high accuracy. Typical receptor-mimicking DNA nanostructures include two transmembrane DNA with a recognition domain, an anchor domain, and an amplification domain. When the signal inputs arrive at the GUV outer membrane, the two transmembrane DNA hybridize with each other and hence enter close spatial proximity. The amplification domain consequently forms a G-quadplex structure or complement strands, resulting in the generation of further chemical reactions inside the GUVs. To date, receptor-mimicking DNA nanostructures capable of detecting ATP,^149^ lysozyme^149^ (Fig. 6(g)), and DNA messengers^150^ (Fig. 6(h)) have been reported.

All the above reports have been achieved during the past decade. Thus, the construction of DNA nanopores and receptor-mimicking DNA nanostructures with more complicated functions is expected to accelerate in the next decade. For DNA nanopores, current studies perform molecular transport using simple diffusion, whereas active transport of molecules is highly desired. For receptor-mimicking DNA nanostructures, systems that can detect multiple molecules and generate multiple reactions simultaneously are yet to be realized.

### Synthetic channels

Synthetic supramolecular complexes have emerged as a type of pore capable of functioning in lipid membranes.^151^ These pores are always smaller than 1 nm in diameter and thus only transport ions, similar to ion channels in living cells. Previous studies have referred to these pores as “nanopores” or “nanochannels”, but without clear criteria. In a distinction from the nanopores that can transport both ions and larger molecules, in this review, we define pores based on synthetic supramolecular complexes as “synthetic channels”. Thanks to the wide chemical repertoire, synthetic channels with different functions have been generated to date. A comprehensive introduction to this field is beyond the scope of this review, so we will focus on the recent advances in this field and discuss how they can impact the field of molecular robots (for a full survey of synthetic channels, we direct the readers to previous reviews^31,152–154^).

**Fig. 7:**
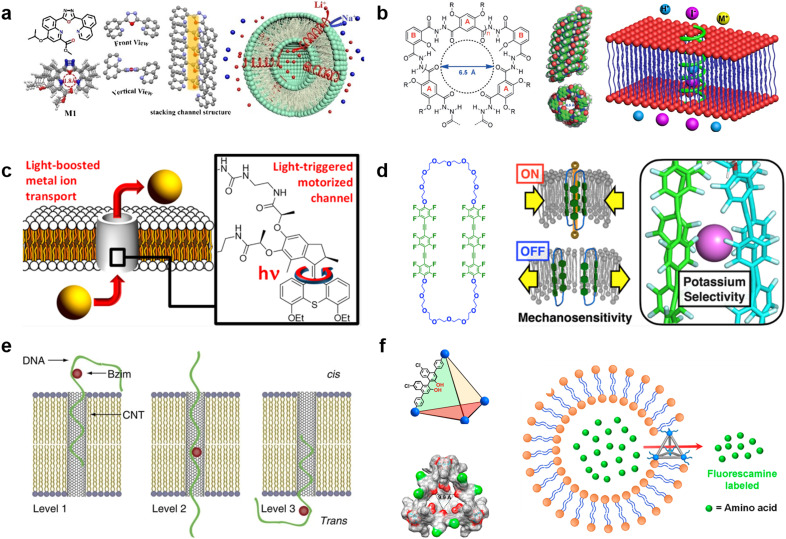
Synthetic channels for transmitting information. a, Chemical structures of aromatic molecules (left). Single crystal structure of aromatic molecules and its linearly self-assembling channel structure (central). Illustration of selective transmembrane transport of Li^+^ (right).^163^ b, Chemical structure of foldamer (left). Side and top view of the foldamer-based synthetic channel. Illustration of selective transmembrane transport of I^−^ (right).^161^ c, Schematic showing light-driven transport of metal ions and an enlarged view of the molecular motor.^170^ d, Chemical structure of amphiphilic cyclophanes with perfluorinated aromatic units (left) and its response to mechanical stress with selective transport of K^+^.^172^ e, Illustration of benzoimidazole (Bzim)-modified 5hmC-containing DNA2 translocation through a SWCNT.^181^ f, Illustration and single-crystal X-ray structure of the tetrahedral MOCs (left). Transport process of amino acids through the tetrahedral MOCs (right).^187^ Figures reproduced with permission from: a, ref. 163, copyright 2023, Wiley-VCH; b, ref. 161, copyright 2020, Wiley-VCH; c, ref. 170, copyright 2021, American Chemical Society; d, ref. 172, copyright 2022, American Chemical Society; e, ref. 181, copyright 2013, Nature Publishing Group; f, ref. 187, copyright 2021, American Chemical Society.

Selective ion transport is the most well-established function for synthetic channels and is a highly desired function for molecular robots because it affords the opportunity for strict regulation of information input. Similar to natural ion channels,^155^ the selectivity of synthetic channels is highly dependent on both the pore lumen size and the type of functional groups in the pore lumen. Starting from several ground-breaking works in 1990s,^156–158^ synthetic channels that can selectively transport K^+^,^159^ Cl^−^,^160^ I^−^,^161^ and H_2_O^162^ have been reported. Currently, efforts are focusing on constructing channels that can selectively transport smaller cations.^163–165^ In 2023, Zhang *et al.* reported a Li^+^-selective synthetic channel using self-assembled aromatic molecules with well-adjusted lumen size and coordination numbers^163^ (Fig. 7(a)). The group found that the channel, with a 1.8 Å-diameter lumen and four ion coordination sites inside the cavity, exhibits higher transport selectivity for Li^+^ over Na^+^, achieving Li^+^/Na^+^ selectivity ratios ranging in value from 15.1 to 23.0. Meanwhile, activities in developing new types of anion-selective channels continue unabated.^161,166,167^ In 2020, Roy *et al.* reported a fully hydrogen-bonded, helically-folded aromatic foldamer-based synthetic channel, with a central lumen rendered by the many methyl groups^161^ (Fig. 7(b)). The methyl groups provide a positive electrostatic potential, resulting in a selectivity towards anions over cations. Moreover, I^−^ was observed to be more favourably transported than Cl^−^ due to having fewer hydrogen bonds than Cl^−^ (I^−^/Cl^−^ selectivity ratio was around 11).

Responsivity to external stimuli is another essential function for synthetic channels and would provide the ability for remote-control in molecular robots. Light is one of the most widely used stimuli due to having high biocompatibility and ease of spatio-temporal control. So far, light-responsive synthetic channels which are irreversibly^168^ and reversibly^169^ photo-controlled have been developed, and recent studies focus predominantly on reversible photo-control. In 2021, Wang *et al.* reported a light-driven synthetic channel that can actively transport alkali ions^170^ (Fig. 7(c)). To achieve active transport, the group included a light-driven rotary motor around the channel. Under light irradiation, the motor provides thermal energy to the channel to help overcome the activation barrier necessary to translocate ions between macrocycles, leading to an increase in Na^+^ transport activity (up to 400%). In living cells, mechanical stress is a major stimulus involved in the regulation of structure and function. In the cytoplasmic membrane, ion channels called mechano-sensitive channels (MSCs) sense mechanical stress and enhance substance influx/efflux as an output. Synthetic channels that mimic the function of MSCs to respond to mechanical stress are now attracting great attention. The first mechano-sensitive synthetic channel was reported by Muraoka *et al.* in 2017.^171^ Using a channel consisting of repeating oligo-(ethylene glycol) (OEG) and aromatic units, with subunits that destabilize the pore structure, the ion transport activity was observed to decrease with expanding membrane tension. In 2022, the group further extended the concept to develop a synthetic channel with potassium selectivity by using amphiphilic cyclophanes with perfluorinated aromatic units^172^ (Fig. 7(d)), opening up the possibility to process multiple signals with synthetic channels. Other synthetic channels responding to stimuli including voltage^173,174^ and the presence of ligands^175,176^ have also been reported in recent years.

Over the past decades, many efforts have been made to construct synthetic channels employing macrocyclic molecules,^177^ foldamers,^178^ and π-stack architectures.^171^ Nevertheless, the synthesis of conventional synthetic channels varies case-by-case, making it challenging to prepare channels with different functions using a general protocol, and hence restricting their use in molecular robots. Carbon nanotubes (CNTs), which are all-carbon hollow nanobarrels with a diameter in the range of 0.8–2 nm,^62^ are considered as a versatile synthetic channel that can be easily prepared. Benefiting from a larger size than conventional synthetic channels, previous reports have shown that CNTs can transport not only ions and water molecules,^179^ but also larger molecules such as amino acids^180^ and ssDNAs.^181^ Importantly, Liu *et al.*, utilized CNTs to selectively detect benzoimidazole (Bzim)-modified 5-hydroxymethylcytosine (5hmC) in ssDNA^181^ (Fig. 7(e)), opening the door to the use of CNT-based molecular robots for the diagnosis of gene mutations. Moreover, a recent study by Marcotte *et al.* has shown that CNTs enable mechanically-activated ionic transport that depends quadratically on the applied pressure,^182^ making CNTs a potential candidate as mechanical stress sensor.

Metal–organic polyhedra (MOPs), also known as metal–organic cages (MOCs), have emerged as another popular option in the synthetic channel family.^183^ MOPs/MOCs are nano-sized cages constructed by linking transition metal clusters with organic ligands, hence benefiting from the ability to control functionality by choosing appropriate metal clusters and ligands from a wide variety of options.^184^ The first report that studied the interaction between MOPs/MOCs with lipid membranes was by Jung *et al.* in 2008, and their results suggested that MOPs/MOCs synthesized from 5-dodecoxybenzene-1,3-dicarboxylic acid (5-OC_12_H_25_-mBDCH_2_) and Cu(CH_3_CO_2_)_2_·H_2_O have transport selectivity dependent on cation size.^185^ In 2017, Kawano *et al.* synthesized rhodium metal–organic polyhedrals (RhMOPs) and demonstrated their multiple conductance states, which are proposed to result from reversible geometry switching of the RhMOPs, between square and triangular geometry of the apertures exposed to the aqueous phase in the lipid membranes.^186^ In 2021, Li *et al.* developed tetrahedral MOCs with hydrogen bonding units inside the cage cavity using six chiral BINOL-derived ligands and four *n*-Bu_3_-Cp_3_Zr_3_ clusters, and demonstrated their ability to transport amino acids^187^ (Fig. 7(f)). Importantly, the chiral cavity enables enantiospecific recognition of amino acids, which is a rarely-reported function for conventional synthetic channels. The endless potential combinations of metal clusters and ligands make MOPs/MOCs sensors for molecular robots an exciting prospect with limitless possibilities.

## Computers of molecular robots

Molecular robots go hand-in-hand with biological computers, where biological computers take available inputs and translate them into appropriate outputs. Thanks to advances in synthetic chemistry and biology, biologically derived molecules such as DNA and proteins have become readily available, leading to the development of biological computers such as DNA computing systems and CFPS.^188,189^ In this section, we will briefly explain the characteristics and reveal the research trends in DNA computing and CFPS.

### DNA computing

**Fig. 8:**
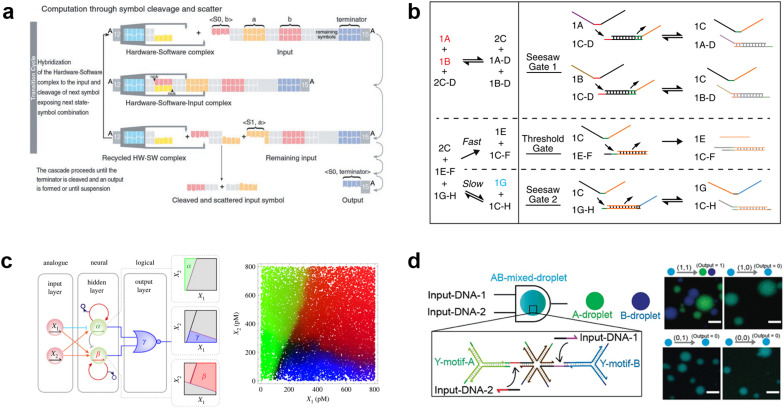
DNA computing for signal processing. a, Suggested mechanism of operation of the automaton by DNA-based state transition using the restriction nuclease FokI and ligase (right).^193^ b, “Seesawing”, “thresholding”, and “reporting” DNA reactions.^360^ c, The decision architecture (left) of the neural network. Fluorescence levels of α, β and γ, measured in approximately 25 000 droplets (right).^361^ d, Illustration of the AND gate operation using DNA droplets (left). CLSM images for droplet phase separation corresponding to four input patterns (right). Scale bars: 10 μm.^212^ Figures reproduced with permission from: a, ref. 193, copyright 2003, National Academy of Sciences; b, ref. 360, copyright 2012, Elsevier; c, ref. 361, copyright 2023, Royal Chemical Society; d, ref. 212, copyright 2022, Wiley-VCH.

Due to the remarkable programmability of DNA molecular behavior based on sequence-dependent hybridization, enzymatic reactions, and strand displacement reactions, DNA computing has emerged as a promising candidate for the computational machinery of molecular robots. The genesis of DNA computing can be traced back to Adleman's pioneering work: massively parallel computation using artificially sequence-designed DNA.^12^ He encoded the nodes and paths in the Hamiltonian path problem onto different ssDNA to execute hybridization-based parallel exploration of the correct Hamiltonian path. By exploiting DNA self-assembly, this methodology allowed large-scale computations to be performed with low energy consumption. Besides the subsequent implementation of mathematical computations (satisfiability problem,^190,191^ maximal clique problem,^191^*etc.*), Benenson *et al.* constructed DNA-based finite automata with two states using programmed DNA sticky/blunt ends, restriction nuclease, and ligase^192^ (Fig. 8(a)). The two states in the automata run at a rate of 109 transitions per second in an input-responsive manner, being the prototype of the smallest biocomputer^193^ as certified by Guinness World Records. Originating from the above single-information processing systems, the development of DNA computing has recently steered towards multiplex information processing.

As a multi-input responsive module, logic gate operations were adopted due to the inherent simplicity in harnessing binary information represented by ‘0’ and ‘1’. Stojanovic *et al.* first demonstrated a DNA-based logic gate using deoxyribozymes (DNAzymes).^194^ In their pioneering approach, two ssDNA were defined as inputs and a DNAzyme was employed as a computational module to catalytically cleave DNA in a logically controlled manner, producing a distinct output that was detected *via* fluorescence readout. Based on this principle, they successfully implemented NOT, AND, and XOR gates. This was followed by other groups that reported the construction of OR, NOR, and NAND gates using DNAzyme/ribozyme-based methodology.^195,196^

Having developed basic logic gates, the next phase of the research effort focussed on cascading the individual gates towards the construction of DNA circuits. DNA strand displacement has emerged as a pivotal technology for implementing cascades, providing a more permissive operating framework than the use of DNAzymes/ribozymes that require strict experimental conditions.^197^ In strand displacement reactions, an input ssDNA reacts with a double-stranded DNA (dsDNA), where it selectively binds to an exposed single-stranded region. This binding event leads to the displacement and release of a pre-existing hybridized ssDNA component. In 2006, Seelig *et al.* proposed a way to cascade DNA logic gates using strand displacement reactions.^198^ In their approach, the output ssDNA released by the strand displacement reaction in the first layer gate was exploited as a subsequent input for the next layer gates. By programming short oligonucleotides to bind/release and incorporating fluorophore/quencher modification, AND, OR, and NOT gates were cascaded without any enzymatic reactions. The same group then extensively applied this strand displacement principle to construct a larger-scale circuit consisting of AND and OR gates using 130 DNA strands, with a combination of “seesawing”, “thresholding”, and “reporting” DNA reactions^199^ (Fig. 8(b)). Importantly, the group demonstrated the circuit's ability to compute the floor of the square root of a four-bit binary number, enabling digital logic networks to be compiled into DNA-based implementations. Moreover, circuits with unique functions, for example, DNA circuits with enhanced reaction speed, responsiveness, and robustness using the cationic polymer, poly(l-lysine)-*graft*-dextran,^200^ in addition to temporal DNA circuits that can respond to both the presence and history of a molecular environment,^201^ and pH-responsive switchable DNA circuits^202^ have all been reported. Through such cascading, scaling, and additional functionalization, DNA circuits can potentially attain the capabilities of reliable computation, precise error correction, and automated circuit compilers, ultimately achieving a computational behaviour reminiscent of electronic computers in wet biological environments.

Besides the binary information processing as described above, what types and level of complex information can DNA computing handle? One intriguing topic is the construction of neural networks, mathematical models that mimic the interconnected neurons of the human brain, using DNA molecules. Qian *et al.* demonstrated the translation of arbitrary linear threshold circuits (neural network model) into strand displacement cascades as a Hopfield network consisting of four interconnected artificial DNA-based neurons.^203^ By setting the proper weights and thresholds for each neuron, the network was able to ‘remember’ a series of binary patterns, opening up possibilities for embedding ‘learning’ into DNA computations. Following this remarkable demonstration, several types of neural networks with training processes were constructed such as winner-take-all neural networks that recognized 9 patterns from 100-bit inputs,^204^ convolutional neural networks that recognized 32 categories from 144-bit inputs,^205^ and oscillation-based reservoir computing as a type of recurrent neural network.^206^ Focusing on the practical applications of DNA-based neural networks, decision-making functionality is useful for clinical diagnosis in healthcare. Okumura *et al.* recently proposed nonlinear decision-making using DNA-based enzymatic neurons with adjustable weights and biases. Combining a hidden layer with two linear classifiers and a logical layer with a NOR gate, the network correctly partitioned the concentration space of the two cancerous miRNAs (X1 and X2) as inputs into three nonlinearly separable regions using distinct fluorescence (α (green), β (red) γ (blue))^207^ (Fig. 8(c)). As represented in this report, oligonucleotide biomarkers, such as miRNA, can be used as inputs in DNA computation. Diagnostic applications are therefore an emerging trend in the development of medical DNA computation,^208,209^ holding the promise of substantial impacts on the advancement of medical molecular robotics. Furthermore, the above DNA-based neural network architectures offer the fascinating prospect of endowing molecular robots with ‘intelligence’, including the realisation of intricate human-brain-like functionalities from perception to memory and consciousness, using DNA.

In addition to the liquid-phase DNA computations described so far, other computational methods using DNA droplets/condensates have also attracted attention. For instance, the Takinoue group developed DNA droplets based on liquid–liquid phase separation of Y-motif DNA consisting of three ssDNA, whose sticky ends enabled selective and exclusive fusion of the droplets.^210,211^ Based on this DNA droplet technology, the group further developed computational DNA droplets with AND gate functionality, where the mixed droplet separated into two distinct droplets only in the presence of two input DNA (= input (1, 1))^212^ (Fig. 8(d)). Moreover, using cholesterol modification as an alternative to DNA sticky ends, the Di Michele group established DNA condensates with a responsive core-shell structure^213^ and with internal domains induced by reaction–diffusion waves.^214^ These technologies can be integrated with GUVs-based molecular robots as components with input-responsive computational capabilities.

### Cell-free protein synthesis (CFPS)

The current developments in the field of molecular robots have focused on using DNA as the output for the computational machinery. On the other hand, the diverse building blocks of proteins make them an attractive alternative output, capable of increased information relay compared with DNA. To this end, another promising tool by which molecular robots respond to external stimuli and convert these into signals or outputs is CFPS. CFPS is capable of synthesizing proteins in a test tube *via* transcription and translation from DNA, without the use of living cells.^215,216^ CFPS can be divided into two types: a reconstituted system and a cell-extract-based system. A reconstituted system is a CFPS where components essential for protein synthesis are highly purified individually and reconstituted in a test tube to carry out the reactions, while the cell-extract-based system is essentially a crude cell extract that contains not only the protein translation machinery but also additional molecules involved in other reactions. A reconstituted system has the advantage of less contamination by factors unrelated to protein synthesis and the possibility of tailoring the composition of the reaction on demand. For these reasons, not all but many of the molecular robots consisting of CFPS reported to date use a reconstituted system, the PURE system,^14^ with its high adjustability.

**Fig. 9:**
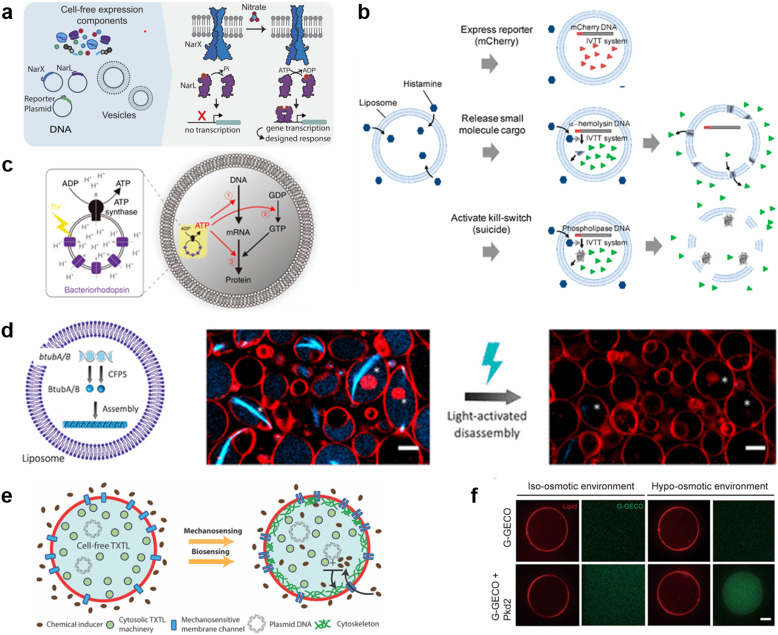
Cell-free protein synthesis system (CFPS) DNA for signal processing. a, Using a two-component sensing system (NarX–NarL) to sense various substances.^217^ b, Controlling the fate of GUVs with a histamine-responsive riboswitch.^219^ c, Synthesis of a reporter protein using light irradiation of ATP synthase and bacteriorhodopsin-containing GUVs.^223^ d, Deforming GUVs from elliptical to spherical shape by degrading CFPS synthesized BtubA/B formed microtubes into monomers using light irradiation. Scale bars: 5 μm.^224^ e, Expression of a bacterial cytoskeletal protein with GUVs loaded with CFPS and the mechanosensitive protein MscL by exposing the GUVs to hypo-osmotic solution.^225^ f, Activating the CFPS-synthesized ion channel Pkd2 with osmotic stress on the membrane to enhance the influx of calcium ions. Scale bars: 10 μm.^226^ Figures reproduced with permission from: a, ref. 217, copyright 2023, National Academy of Sciences; b, ref. 219, copyright 2019, American Chemical Society; c, ref. 223, copyright 2020, Nature Publishing Group; d, ref. 224, copyright 2021, American Chemical Society; e, ref. 225, copyright 2019, American Chemical Society; f, ref. 226, copyright 2022, American Society for Cell Biology.

Input signals for controlling the fate of molecular robots consisting of CFPS can be divided into two major categories: 1) chemical and 2) physical signals. In response to the input of various chemical signals, intravesicular CFPS has been shown to synthesize proteins, most often reporter proteins. Kamat's group has developed a sensor that expresses luciferase in response to nitrate.^217^ In the bacterial two-component sensing system, NarX–NarL, the binding of nitrate to the sensor domain of NarX induces phosphorylation of NarL by the kinase domain of NarX. The phosphorylated NarL acts as a transcriptional activator and downstream reporter proteins are expressed (Fig. 9(a)). The authors reconstituted the NarX–NarL system inside GUVs and showed not only the sensing of nitrate but also that of nickel, iron, and vancomycin is possible, by only modifying the sensor module. In addition, the same group has recently shown the use of a fluoride-responsive riboswitch that allows detection of the presence of fluoride in the environment by coupling it to the expression of reporter proteins.^218^ While the examples above use previously reported or natural response elements, Dwidar *et al.* started by creating a novel riboswitch. They created a histamine-responsive riboswitch using SELEX. The riboswitch was encoded upstream of a reporter protein, pore-forming protein, or phospholipase, which allowed the GUVs to show fluorescence, release small chemicals, or self-destruct, respectively^219^ (Fig. 9(b)). Other examples of chemical signalling control over molecular robots include membrane deformation of GUVs by external supply of FtsZ synthesized by CFPS,^220^ a fusion of two different vesicles by calcium and polyethylene glycol to initiate protein synthesis inside GUVs,^221^ and driving an in-chip and lipid-compartmentalized protein synthesis using CFPS by supplying an energetic substance to the outside of the lipid membrane.^222^

In addition to chemical signalling, light and osmotic stress have been used as physical input signals. Kuruma's group synthesized F0F1 ATP synthase and bacteriorhodopsin using CFPS and incorporated both into small lipid vesicles to prepare energy-generating proteoliposome (PL). Upon light irradiation, bacteriorhodopsin created a proton gradient across the membrane, which then was used by ATP synthase for the phosphorylation of ADP to ATP. The PL encapsulated inside the GUVs was used as an ATP regeneration system and using the generated ATP, GFP synthesis was performed with CFPS^223^ (Fig. 9(c)). Danelon's group showed the synthesis of BtubA/B microtubule formation inside of GUVs, which ultimately deformed GUVs from spherical to elliptical shapes. Light irradiation cleaved the microtubes into monomers and restored the spherical shape of GUVs^224^ (Fig. 9(d)). When MscL, a mechanosensitive protein, was synthesized using CFPS inside GUVs and osmotic stress was applied, MscL changed its conformation and opened a pore, resulting in the influx of an inducer which then triggered the expression of a bacterial cytoskeletal protein^225^ (Fig. 9(e)). Another example synthesized Pkd2, an ion channel, inside GUVs and upon an increase in osmotic stress, an influx of Ca^2+^ ions was observed *via* the binding of the ion with its indicator G-GECO^226^ (Fig. 9(f)).

As described above, molecular robots, in response to chemical and physical inputs, have exhibited various outputs using CFPS as a computer. For chemical input signals, the permeability of the chemicals plays an important role. Membrane-permeable chemicals can directly affect the CFPS inside the GUVs, whilst membrane-impermeable chemicals require receptors or transporters to transmit the chemical signal to the intravesicular CFPS. Several examples have been reported,^217,227^ however, since many input signalling molecules are charged and have little membrane permeability, the development of a sensing domain using receptors or transporters is a future challenge. For physical inputs, temperature shift, pH change, redox state, and electron transfer are yet to be realized. As for the outputs, many studies are limited to the expression of reporter proteins. These are sufficient as proof-of-concept experiments or sensor developments. However, more sophisticated output including motility and cooperative interaction with extravehicular environments are yet to be realized.

## Actuators of molecular robots

Molecular robots that achieve complicated tasks are desired to exhibit specific physical movements such as deformation and propulsion. So far, we have demonstrated the power of DNA and proteins as molecular tools to construct sensors and computers for molecular robots. In a similar vein, DNA and proteins also provide a unique path forward in building actuators for molecular robots.^228,229^ This section aims to give a brief overview of the strategies to actuate molecular robots.

### DNA nanotechnology

DNA nanotechnology enables exquisite control over the structure of self-assembled macromolecular and nanoscale motifs.^230^ DNA nanodevices are however far from static and have been engineered to reconfigure, change shape, and move in response to a wide array of stimuli.^230,231^ From the ground-breaking examples of DNA tweezers^197^ and walkers,^232^ to the origami crank-sliders and joints demonstrated by the Castro group,^233^ to the bioinspired rotors built by the Dietz and Simmel groups,^234,235^ DNA nanomachines and nano-actuators have demonstrated an unparalleled ability to control different types of motion at the nanoscale. Furthermore, these nanodevices can be actuated through a variety of different stimuli, from strand displacement^236^ to the species and concentration of cations,^237,238^ to changes in pH,^237^ light exposure,^239^ and enzymatic action.^240^

As discussed in the section on DNA nanopores, DNA nanostructures can be mechanically coupled to lipid membranes using lipophilic anchors, typically cholesterol or tocopherol.^241,242^ This coupling unlocks vast opportunities to engineer both the morphology and dynamic responses of GUV-based microrobots and artificial cells through membrane-anchored DNA devices that imitate the functions of membrane proteins.

**Fig. 10:**
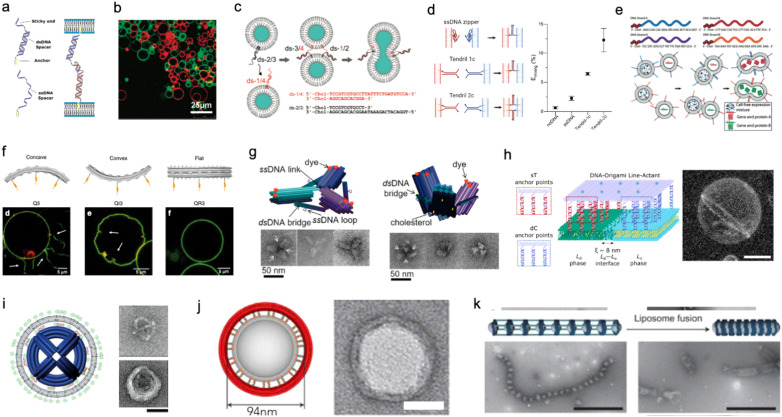
DNA nanostructures for actuating lipid membranes. a, Schematic of DNA linkers anchored to membranes and mediating adhesion between lipid vesicles. Linkers may feature a double-stranded DNA spacer (top) or be fully single-stranded (bottom).^242^ b, Confocal micrograph of GUVs adhering due the action of single-stranded DNA linkers (right), adapted from ref. 247. c, DNA “zipper” constructs mediating lipid vesicle fusion.^252^ d, Comparison of fusion efficiency (right) from various DNA zipper and “tendril” constructs (left).^255^ e, Cascades of lipid vesicle fusion reactions mediated by DNA zippers trigger CFPS.^256^ f, Curved DNA origami (top) influence the morphology of GUVs as determined with confocal microscopy (bottom).^261^ g, Clathrin-like DNA origami triskelia of controllable curvature (top) and their TEM images (bottom).^262^ h, DNA origami lineactants (left) accumulate at the interface between liquid ordered and liquid disordered domains in phase-separated GUVs (confocal projection, right) thanks to the phase-selectivity of double cholesterol (dC) and single tocopherol (sT) anchors. Scale bar: 10 μm.^271^ i, Virus-like DNA particle obtained by templating the formation of a lipid vesicle around a spherical DNA origami decorated with lipids (left) and TEM images of a bare (top right) and lipid-enveloped (bottom right) origami. Scale bar: 50 nm.^272^ j, Lipid vesicles of controlled size templated by lipid-modified DNA-origami rings. Images are TEM micrographs of the constructs shown on the immediate left. Scale bars: 50 nm.^362^ k, Liposomes captured by dynamic DNA origami arrays. Scale bars: 100 nm.^363^ Figures reproduced with permission from: a, ref. 242, copyright 2019, IOP Publishing; b, ref. 247, copyright 2007, American Chemical Society; c, ref. 252, copyright 2008, American Chemical Society; d, ref. 255, copyright 2022, Royal Chemical Society; e, ref. 256, copyright 2019, Wiley-VCH; f, ref. 261, copyright 2018, Nature Publishing Group; g, ref. 262, copyright 2019, American Chemical Society; h, ref. 271, copyright 2023, American Chemical Society; i, ref. 272, copyright 2014, American Chemical Society; j, ref. 362, copyright 2017, Elsevier; k, ref. 363, copyright 2020, Wiley-VCH.

Membrane adhesion is among the most basic functions mediated by cell-surface receptors, underpinning a plethora of biological processes, including motility, tissue formation, mechanosensing, and endocytosis. Exploiting the selectivity of base-pairing interactions, synthetic, membrane-anchored DNA linkers have been used to induce and program adhesion between lipid membranes^242^ (Fig. 10(a)), starting with the seminal works of Höök and coworkers,^243,244^ Boxer and coworkers^245,246^ and Beales and Vanderlick^247,248^ (Fig. 10(b)). Parolini *et al.* have then demonstrated the DNA-mediated assembly of thermoresponsive synthetic tissues^249^ and, leveraging toehold-exchange reactions, established control over the kinetics of tissue formation.^250^

Membrane fusion is another ubiquitous mechanism that living cells use to manipulate the structure and composition of their lipid membranes, manifesting, for instance, in neurotransmitter release from synaptic vesicles and in the invasion by enveloped viruses. Dynamic DNA nanostructures have been designed to replicate the response of extant fusogenic protein machinery, notably SNARE proteins.^251^ Höök and co-workers first demonstrated that zipper-like DNA constructs anchored to membranes can induce fusion by bringing the membranes into molecular proximity,^252^ as shown in Fig. 10(c). The efficiency of fusion has been observed to depend on nanostructure design, for instance the presence of non-binding DNA spacers between the lipophilic anchors and the zipping domains, as well as on the number and chemistry of the hydrophobic anchors and the surface density of the DNA-zippers.^253,254^ Later designs have included dsDNA “tendrils”, where the fusogenic zipping action is mediated by four-way branch migration, which were found to improve fusion efficiency and facilitate its modulation with soluble DNA strands^255^ (Fig. 10(d)). Membrane composition can also significantly influence fusion efficiency,^252,253^ which increases substantially in the presence of conical lipid species (*e.g.* DOPE)^255^ and for phase-separated membranes.^256^ Fusogenic DNA constructs have been applied to engineer complex behaviors in artificial cell systems, for instance triggering CFPS^256^ (Fig. 10(e)) and mediating intricate fusion pathways that mimic cell differentiation.^257^ Such nanodevices have also been shown to facilitate fusion between synthetic and biological cells for the purpose of intra-cellular delivery^258^ or to generate “hybrid” cells.^259^

Similar to smaller nanostructures, DNA origami can be linked to membranes *via* multiple hydrophobic moieties.^260^ Owing to their larger size, stiffness, and precisely programmable 3D geometry, membrane-bound origami can be used to influence the morphology of synthetic lipid membranes, mimicking membrane-sculpting proteins. Czogalla *et al.* first demonstrated that, for sufficiently high coverage, polymerizable, brick-like DNA origami can induce large-scale deformation in GUVs, which were found to lose their equilibrium spherical shape.^260^ Franquelim *et al.* later showed that GUVs decorated with convex origami imitating natural bar-domain proteins, acquired shallow invaginations upon osmotic deflation.^261^ In turn, functionalization with concave origami induced tubulation, which became more prominent for increasing origami coverage^261^ (Fig. 10(f)). Following a similar principle, Journot *et al.* designed convex DNA-origami “triskelia” resembling clathrin proteins. The origami triskelia could be polymerized to form a two-dimensional network, producing membrane deformation reminiscent of clathrin-coated invaginations^262^ (Fig. 10(g)).

Membrane-adhesion and the polymerization state of DNA nanostructures can be externally controlled through a variety of different mechanisms, including strand displacement reactions,^263,264^ pH-responsive non-canonical DNA constructs,^265^ light-responsive azobenzene-modified DNA linkers,^266^ and by modulating ionic composition and membrane phase.^267^ These tools open up vast opportunities for controlling dynamic reconfiguration in lipid-based microrobots and engineering biomimetic pathways for environmental adaptation.

The differences in affinity that different lipophilic anchors display for co-existing lipid phases also offer a useful mechanism for programming dynamic responses in DNA-functionalised synthetic membranes.^268,269^ Rubio-Sánchez *et al.* considered phase-separated GUVs with liquid-ordered (Lo) and liquid disordered (Ld) domains and decorated them with DNA nanostructures using both double-cholesterol (dC) and single-tocopherol (sT) anchors, which display a preference for Lo and Ld, respectively.^270^ The authors demonstrated that connecting and disconnecting selected anchors *via* strand displacement could induce the re-distribution of the DNA nanostructures on the surface of the GUVs – a simple example of directed cargo transport. Specifically, linking dC and removing sT would cause the devices to accumulate in the Lo phase, while removing the dC and bringing back the sT would trigger migration to Ld.^270^ In a subsequent contribution, Rubio-Sánchez *et al.* noted that DNA origami plates functionalized with both dC and sT, arranged at opposite ends of the plate, showed the tendency to accumulate at the line-interface between Lo and Ld (Fig. 10(h)). These DNA-origami line-actants (DOLAs) were shown to reversibly stabilize small lipid domains against coalescence, establishing a route to control the surface patterning of GUVs. DOLAs were also shown to provide control over a biomimetic membrane-fission pathway, whereby budding-off of DOLA-stabilised domains could be induced upon de-activating the line-actants under hyperosmolar conditions^271^ (Fig. 10(h)).

When the overall size of the lipid vesicles one seeks to engineer approaches the nanoscale, DNA origami can be used to directly template the formation of vesicles with precisely controlled size and shape. This concept was first proposed by Perrault and Shih, who used spherical, lipid-modified DNA-origami to template the formation of monodisperse lipid vesicles (diameter ∼76 nm)^272^ (Fig. 10(i)). The lipid vesicles would form around the origami following lipid addition and removal of an initially present surfactant. The lipid envelope was found to protect the DNA nanostructure from enzymatic degradation – a useful feature for envisaged *in vivo* deployment.^272^ A similar strategy has been pursued by Yang *et al.* using DNA-origami rings of various diameters that, thanks to a lipid-modified inner edge, could template the growth of size-controlled lipid vesicles in a Saturn-ring-like geometry^273^ (Fig. 10(j)). Zhang *et al.* later demonstrated that similar DNA-origami exoskeletons could be polymerized, generating arrays of lipid vesicles with controlled inter-membrane distance, or tubular vesicles^274^ (Fig. 10(k)). The devices could also be actuated to induce lipid vesicle fusion into and bending of the lipid tubes.^274^ In addition to providing control over lipid architectures, lipid vesicle-templating DNA origami has also been applied to precisely position molecules onto membranes and study reaction kinetics with absolute control over stoichiometry.^275^

### Protein and peptides

**Fig. 11:**
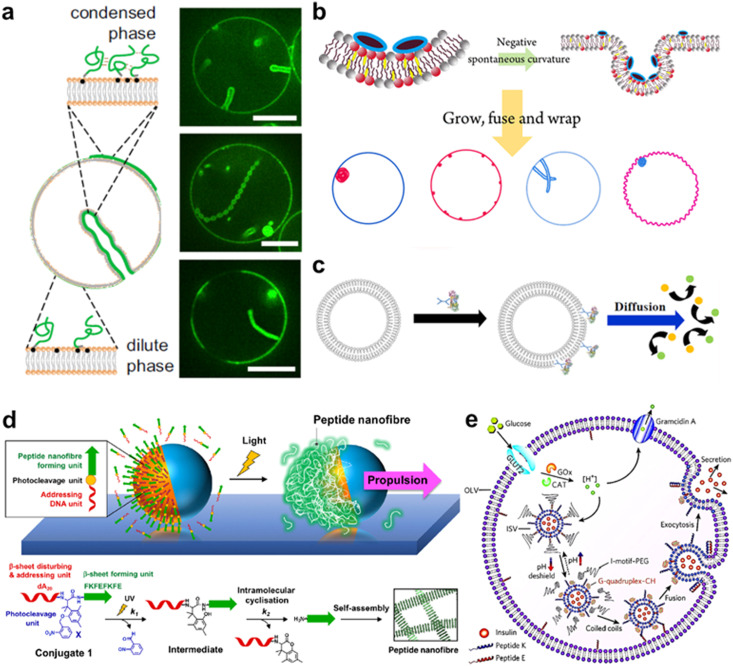
Proteins and peptides for actuating lipid membranes. a, GUV deformation triggered by phase separation of FUS LC adhered on the lipid membrane. Scale bars: 10 μm.^279^ b, GUV deformation triggered by *de novo* peptides.^285^ c, Propulsion of ATPase-coated GUVs.^286^ d, Light-induced propulsion of a phase-separated GUVs driven by local peptide nanofibre growth (top) and the photocleavage reaction of a DNA-peptide conjugate (bottom).^289^ e, Illustration of the biochemical process inside OLVs. Vesicle fusion is triggered by the interaction between peptide K and peptide E.^364^ Figures reproduced with permission from: a, ref. 279, copyright 2021, National Academy of Sciences; b, ref. 285, copyright 2018, American Chemical Society; c, ref. 286, copyright 2019, American Chemical Society; d, ref. 289, copyright 2018, Nature Publishing Group; e, ref. 364, copyright 2021, Royal Chemical Society.

Owing to their high affinity to lipid membranes, proteins and peptides act as promising actuators in molecular robots, in the same way that they deform or propel living cells. Deformation could allow molecular robots to overcome complex terrains. Various proteins/peptides have been reported to deform GUVs.^17,276–283^ Membrane deformation can be classified into two broad categories: outward deformation and inward deformation. Inward deformation is considered more energetically unfavorable because it leads to membrane expansion.^284^ As a consequence, most of the previous studies focus on outward deformation. However, inward deformation is also an attractive route towards molecular robots with increased functionality, such as endocytosis-like membrane fission. In 2021, Yuan *et al.* reported inward bending of the membrane of phase-separated GUVs of membrane-bondable N-terminal low-complexity domain of fused in sarcoma (FUS LC) proteins^279^ (Fig. 11(a)). Meanwhile, Yu *et al.* designed a *de novo* amphiphilic bola-type peptide composed of lysine and leucine that regulates inward budding of lipid membranes^285^ (Fig. 11(b)).

Propulsion could help molecular robots to reach their various destinations. One strategy to perform propulsion is to make use of the diffusive movement of enzymes. In 2019, Ghosh *et al.* showed that GUVs with ATPase-tagged lipid membranes exhibited enhanced mobility in the presence of ATP.^286^ In 2023, Jin *et al.* demonstrated that horseradish peroxidase (HRP)-decorated Janus GUVs undergo directional motion in the presence of H_2_O_2_, while such enhanced mobility is absent from homogeneously enzyme-decorated GUVs^287^ (Fig. 11(c)). Another attempt reported by Kurakazu *et al.* involved GUV propulsion using motor protein flagella isolated from *Chlamydomonas.*^288^ Interestingly, the mobility was further enhanced when the GUVs were coated with annexin. On the other hand, there are no reports of peptide-based GUV propulsion, with the need for peptides to convert chemical energy to kinetic energy.

Morphological change with a combination of proteins/peptides and DNA is also of great interest owing to the high programmability and versatility of DNA. A groundbreaking work by Sato *et al.* reported a system using kinesin-DNA complexes to deform GUVs. The group showed that kinesin can be attached to lipid membranes using a light-triggered DNA displacement reaction, leading to a continuous shape change of the GUVs driven by microtubule sliding on the membrane.^15^ Meanwhile, Inaba *et al.* have developed a system in which propulsion is achieved by partial growth of peptide nanofibers on a phase-separated membrane.^289^ In their system, photocleavable peptide-DNA complexes are specifically conjugated to the lipid-disordered membrane. Upon exposure to light, the peptides were released from the membrane and locally self-assembled into nanofibers, resulting in autonomous GUVs propulsion (Fig. 11(d)).

Unique applications have been realised when combining protein/peptide actuators with multi-compartment architecture, such as vesicles-in-vesicles. Chen *et al.* engineered ‘artificial beta cells (AβCs)’ that sense glucose in solution and release insulin by membrane fusion.^290^ They first prepared insulin-encapsulated inner small vesicles (ISVs) with a membrane composed of lipids, peptide (peptide K), and pH-sensitive polyethylene glycol (PEG) conjugates which detach from the membrane at a low pH environment. A mixture of ISVs, glucose oxidase, and catalase are then encapsulated in outer large vesicles (OLVs) containing a lipid membrane with peptide E (complementary to peptide K) and glucose transporters. When glucose was transported from the outside to the inside of the OLVs, a low pH environment was generated due to the glucose oxidase and catalase reaction. The pH decrease triggered PEG detachment from the membrane, and consequently, the unshielded peptide K formed coiled coils with peptide E, promoting the fusion of two vesicles and the release of insulin (Fig. 11(e)). We anticipate future research to develop in the use of proteins/peptides to form complex structures, fine-tuning of morphology changes, and cooperative interaction with each other.

## Applications of molecular robots

Robots that can extend the reach of human engineering capability in the microscale are in high demand. Compared to micro-sized robots using alloys,^291,292^ oxide minerals,^293,294^ and ceramics,^295^ molecular robots are biocompatible and biodegradable due to their use of biological materials. This also affords versatility in biological environments. At the same time, the large toolbox of sensors, computers, and actuators, as mentioned above, enables them to execute multiple and complicated tasks which are challenging for alternatives such as hydrogel-based microswimmers.^296,297^ Additionally, the leakage issues for such microswimmers are resolved in molecular robots owing to the lipid membrane functionality as a semipermeable barrier. Accordingly, molecular robots have exhibited great potential in various applications as a promising alternative to conventional microrobots. In this section, we will provide some representative examples of emerging state-of-the-art applications of molecular robots.

### Detection of molecules

**Fig. 12:**
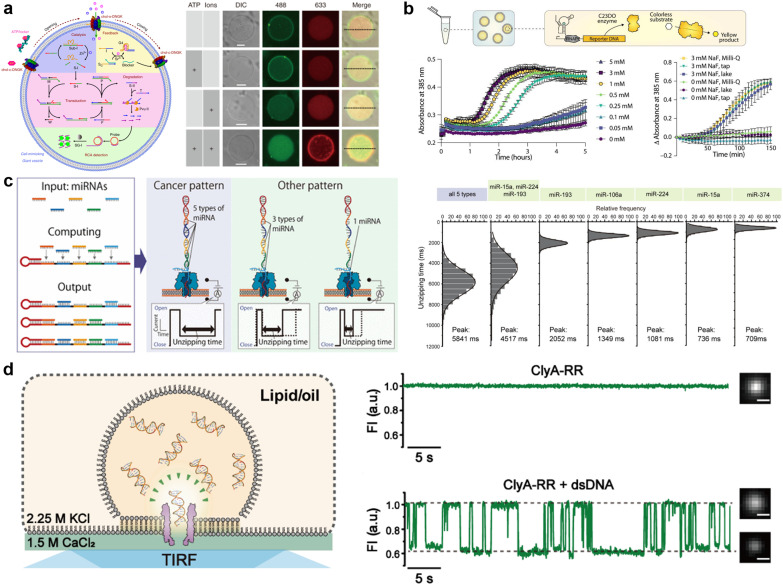
Molecular robots for detection of molecules. a, Detection of Sr^2+^ by GUVs with a DNA nanopore and circuit (left), as observed by the increase of 488 fluorescent signals from SG-I in the presence of ATP and Sr^2+^ (right).^141^ b, Illustration of the GUVs designed to detect sodium fluoride (top) and its absorbance over time in response to *in vitro* samples (bottom left) and real-world samples (bottom right).^218^ c, Using nanopores and DNA computing to detect five types of miRNAs (left) from histograms of the unzipping time of each miRNA pattern (right).^304^ d, Illustration of DiffusiOptoPhysiology (DOP) method (left). Comparison of the signal of Fluo-8 with/without dsDNA in the solution (right).^310^ Figures reproduced with permission from: a, ref. 141, copyright 2020, Nature Publishing Group; b, ref. 218, copyright 2023, AAAS; c, ref. 304, copyright 2022, American Chemical Society; d, ref. 310, copyright 2019, AAAS.

Small toxic molecules (≤1000 daltons), for example, heavy metals and organochlorine, are well-known pollutants in water. Though spectroscopic methods such as inductively coupled plasma atomic emission spectroscopy and mass spectrometry are powerful for molecular detection, the requirement of real-time monitoring of the local molecular concentrations cannot be met with such methods in principle.^298^ On the other hand, molecular robots with sensors and computers may be able to fill this gap. Previous studies have reported that GUVs with nanopores are useful for the detection of K^+^,^56^ Cu^2+^,^299^ and Cl^−^.^300^ Recently, a unique strategy using GUVs with a DNA nanopore and circuit offered by Peng *et al.* suggested switchable detection of Sr^2+^ (ref. 141) (Fig. 12(a)). In the presence of ATP, the DNA nanopore switches from closed to open, which enhances the influx of environmental Sr^2+^. The Sr^2+^ sequentially triggers a set of confined downstream cascade reactions to output both a fluorescence signal and switching of the nanopores back to the closed state. Another pioneering work that uses CFPS inside GUVs to detect fluoride was demonstrated by Boyd *et al.*^218^ In their system, the membrane-permeable sodium fluoride (NaF) initializes the riboswitch-mediated synthesis of catechol (2,3)-dioxygenase (C23DO), which subsequently catalyzes the conversion of its colorless substrate, catechol, to the yellow-colored 2-hydroxymuconate semialdehyde, constituting a colorimetric response (Fig. 12(b)). Importantly, they successfully showed the possibility of detecting fluoride in real-world samples from Lake Michigan and the Evanston, IL municipal tap water supply. Though the sensitivity of molecular robots remains inferior to that of spectroscopic methods, they provide the opportunity to probe in detail the concentration of small molecules in a biological environment. We infer that research into other novel applications, for example, real-time monitoring of the pollutants in aquatic life, is also underway.^301^

Biomarkers such as DNA, microRNAs, and peptides are another essential target for molecular robots. So far, we have reported that nanopores have a strong synergy with DNA computing to allow for highly sensitive detection.^13,302–304^ In our most recent study, we proposed a system for pattern recognition of five types of miRNAs overexpressed in bile duct cancer (BDC) with an αHL nanopore, diagnostic DNA, and a planar lipid membrane^304^ (Fig. 12(c)). With our system, we succeeded in the label-free detection of miRNA expression patterns from the plasma of BDC patients at sub-femtomolar concentrations. On the other hand, detecting peptides using nanopores is more challenging due to peptides' relatively small size and heterogeneous charge compared with polynucleotides. During the last decades, academic efforts have been invested into designing mutant nanopores^305,306^ or optimizing solution conditions^307,308^ to address the above problems. However, there remains a long way before the realisation of peptide detection with amino acid resolution. Meanwhile, biomarker detection with GUV-based molecular robots also likely faces an arduous path to development. Liu *et al.* reported GUVs that detect environmental DNA messengers,^150^ while Barba-Bon *et al.* have used GUVs to monitor the transport of cell-penetrating peptides.^309^ Nevertheless, the sensitivity and amount of information conferred using fluorescence-based detection is several times lower compared with electrical recording technology. Recently, the Huang group has reported an electrode-free nanopore sensing system by a so-called DiffusiOptoPhysiology (DOP) method, which optically monitors fluorescence emission resulting from diffusive binding of Ca^2+^ with its indicator dye Fluo-8 through a nanopore on a planar lipid membrane by total internal reflection fluorescence, and detected dsDNA^310,311^ (Fig. 12(d)). Accordingly, the combination of GUV-based molecular robots with new technology (for example, single-molecule localization microscopy^312^ and stimulated emission depletion microscopy^313^) is worth exploring to facilitate higher levels of detection sensitivity.

### Communication with living cells

**Fig. 13:**
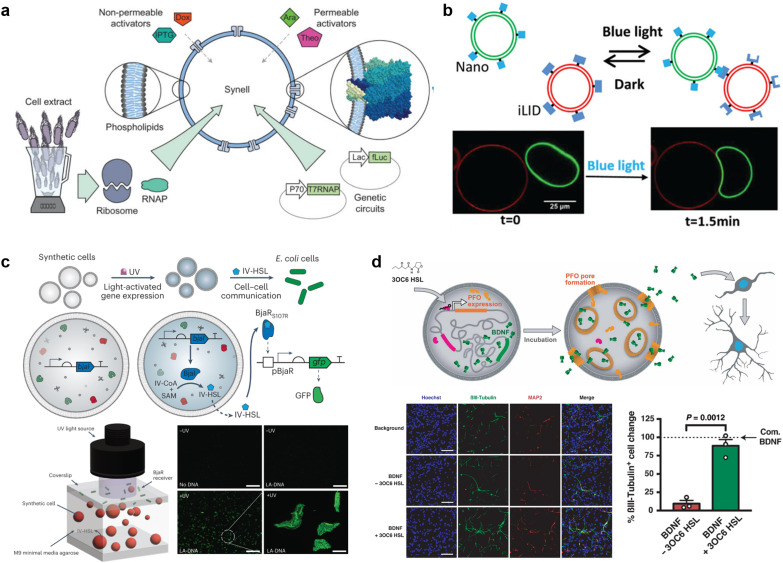
Molecular robots for communication with living cells. a, Modular design of genetic circuit interactions within and between GUVs.^365^ b, Controlling the distance between GUVs with light-oxygen-voltage 2 protein. Scale bar = 25 μm.^326^ c, Schematic representation of communication between GUVs with bacteria (top). GFP is expressed only when the GUVs are exposed to UV light (bottom). Scale bar = 200 μm. Scale bar (bottom right) = 20 μm.^329^ d, Schematic representation of communication between GUVs with neural stem cells (top). GUVs function to enhance stem cell differentiation, as revealed by the increase in the percentage of bIII-tubulin-overexpressing neurons (bottom). Scale bar = 50 μm.^316^ Figures reproduced with permission from: a, ref. 365, copyright 2020, Royal Chemical Society; b, ref. 326, copyright 2019, Wiley-VCH; c, ref. 329, copyright 2023, Nature Publishing Group; d, ref. 316, copyright 2020, AAAS.

Engineering communication between molecular robots and living cells would provide novel strategies to address issues in fields such as drug development or bioremediation. Communication is a process of signal production and transmission. Attempts have been made towards both DNA computing^256,314,315^ and CFPS^316,317^ for signal processing. The use of such systems can greatly improve the complexity and programmability of signalling, endowing molecular robots with the potential of performing complicated tasks. For instance, Adamala *et al.* constructed two populations of lipid vesicles that contain different genetic circuits called “sensor liposomes” and “reporter liposomes”.^317^ When the membrane-permeable arabinose (Ara) was applied to the bulk solution, αHL expression started in the sensor liposomes, consequently enabling the release of the non-membrane-permeable activator β-d-1-thiogalactopyranoside (IPTG). The IPTG was then recognized by the reporter liposomes, triggering the output of luciferase (Fig. 13(a)).

Signal transmission of molecular robots can be accomplished by nanopores including αHL,^317–320^ melittin,^321,322^ and perfringolysin O (PFO).^316^ Importantly, the nanopores not only facilitate the transmission of signals but can also cause leakage problems of other encapsulated molecules. For this reason, heptakis(2,3,6-tri-O-methyl)-beta-cyclodextrin (TRIMEB), which is noncovalently bound into the lumen of αHL, is sometimes introduced to prevent the leakage of encapsulated content.^319,320^ Meanwhile, considering the approximation diffusion equation *t* = *L*^2^/2*D* (here, *t* is the elapsed time since signal transmission began, *L* is the distance from the GUVs to the target, and *D* is the diffusion coefficient), the distance between the GUVs also plays an important role in signal transmission. To this end, significant advances have been made in recent years to engineer membrane adhesion with DNA nanostructures^323^ and proteins.^319,324,325^ Notably, the Wegner group showed that membrane adhesion could be controlled with the use of an improved light-induced dimer protein based on the light-oxygen-voltage 2 (LOV2) domain from *Avena sativa*, opening up a new horizon for designing efficient communication networks^319,325,326^ (Fig. 13(b)).

The above progress has paved the way for the realization of communication between molecular robots and living cells. Quorum sensing, which is the communication behavior of cells to detect and respond to cell population density, has been extended to molecular robots and bacterial populations.^327–329^ Remarkably, Smith *et al.* reported light-activated GUVs that could perform quorum-sensing-based communication with bacteria^329^ (Fig. 13(c)). The system is achieved by installing the T7 promoters of DNA templates with photocleavable biotinylated (PCB) and monovalent streptavidin (mSA) that could impede T7 RNA polymerase from binding to the T7 promoter. When exposed to UV light, the mSA was liberated, resulting in the expression of the BjaI enzyme followed by the production of *N*-isovaleryl-l-homoserine lactone (IV-HSL) in GUVs. As the bacterial populations contained a gene circuit highly sensitive to IV-HSL, they responded to the IV-HSL signal with resultant GFP expression. This work sets the stage for the development of a remote-controlled molecular robot for communication with living cells. Meanwhile, communication between molecular robots and mammalian cells provides another area of interest. A remarkable work by Kaneda *et al.* reported that calcien dye could be transferred from Connexin43 (Cx43)-expressing GUVs to cultured Cx43-expressing U2OS cells through the gap junction.^330^ Recently, Toparlak *et al.* demonstrated GUVs that can drive mammal neural differentiation.^316^ The GUVs contained transcription-translation machinery and DNA templates that coded for brain-derived neurotrophic factor (BDNF), LuxR, and PFO. In the presence of *N*-3-oxohexanoyl homoserine lactone (3OC6 HSL)–responsive transcriptional repressor LuxR, PFO was expressed and assembled into nanopores in lipid membranes, enabling the release of BDNF. After 19-day coincubation with mouse embryonic stem cell-derived neural stem (mNS) cells, the group successfully observed the capability of GUVs to enhance the neural differentiation and maturation, with an increase in pan-neuronal and mature neuronal markers βIII-tubulin and microtubule-associated protein 2 (MAP2) (Fig. 13(d)). We anticipate that future efforts will be geared toward adapting multiple molecular robots and living cell populations to build communication networks with higher complexity.

### Conversion of energy

**Fig. 14:**
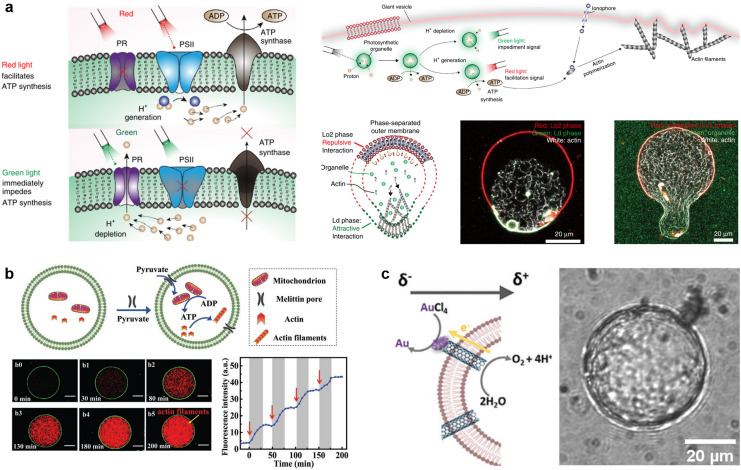
Molecular robots for conversion of energy. a, Facilitating or impeding the ATP synthesis with different light color exposure. (left) Optical stimulation couples ATP synthesis with ATP-dependent actin polymerization and morphological change of the GUVs (top right). Confirmation of the formed cytoskeletal proteins and their impact on membrane deformation with microscopy (bottom right). Scale bar = 20 μm.^366,367^ b, Mitochondrion-containing GUVs' response to pyruvate addition (top). Time-dependent process of cytoskeletal proteins polymerization triggered by the addition of pyruvate (bottom). Scale bar = 10 μm.^333^ c, Schematic showing polarization of carbon nanotubes within lipid membranes, triggering the reduction of gold chloride to solid gold deposits (left). Observed deposition of gold on GUVs (right). Scale bar = 20 μm.^334^ a, ref. 366, copyright 2023, Frontiers Media and ref. 367, copyright 2020, Wiley-VCH; b, ref. 333, copyright 2022, Wiley-VCH; c, ref. 334, copyright 2022, Wiley-VCH.

The operation of sensors, computers, and actuators in molecular robots requires energy sources, hence ATP is sometimes encapsulated in GUVs or added to the external solution.^141,316^ Moreover, such molecular robots may also provide solutions to global energy issues. In a living organism, energy production is powered by a transmembrane proton gradient, meaning that the membrane-spanning ATP synthase turns ADP into ATP only when there is a proton gradient.^331^ In thylakoids, this proton gradient is caused by a light energy-driven proton pump. Accordingly, it has attracted the interest of scientists to incorporate ATP synthase and proton pumps into GUVs to build a self-fuelling system. A groundbreaking work in this field was reported by Lee *et al.* in 2018.^332^ The group built an artificial organelle containing ATP synthases and two light-driven proton pumps, photosystem II (PSII) and proteorhodopsin (PR), and encapsulated it into GUVs. The PSII is a proton pump that can be activated by red light, whereas PR proton pumping is mainly initiated by green light. Moreover, due to the pH-dependent bidirectional proton-pumping ability of PR, PR and PSII work in conjunction to increase the proton gradient at low pH, but counteract the action of PSII at high pH. This makes it possible to facilitate or impede ATP synthesis with exposure to different colors of light. The ATP-dependent polymerization of cytoskeletal proteins was used to confirm ATP synthesis. As expected, the red light facilitated and the green light impeded the growth of cytoskeletal proteins, responding to the operation of PR and PSII. Moreover, the polymerized cytoskeletal proteins showed the ability to deform GUVs with an adjusted lipid composition (Fig. 14(a)). A similar idea was demonstrated by Berhanu *et al.*^223^ Instead of cytoskeletal protein reaction, successful ATP synthesis was monitored by GFP expression in a CFPS.

In eukaryotic cells, energy conversion mainly occurs in the mitochondria. Li *et al.* encapsulated mitochondria extracted from C6 glioma cells into GUVs and showed ATP synthesis with the addition of pyruvate which is a trigger molecule to stimulate mitochondria to produce ATP^333^ (Fig. 14(b)). Though this work paves the way to develop mitochondria-based self-fuelling systems in GUVs, it remains difficult to construct an artificial mitochondrion from scratch, due to the complexity of the citric acid cycle and the electron transport chain. Another fascinating work demonstrated by Hicks *et al.* inserted carbon nanotubes in GUVs to mimic voltage-dependent anion-selective channels, which were able to transfer electrons *via* redox upon a change in potential.^334^ The group showed that a 1.5 V voltage stimulation triggers the reduction of gold chloride to solid gold deposits on the carbon nanotube terminus (Fig. 14(c)). This work opens up the potential for nanopore transistors, able to convert electrical energy to chemical energy in molecular robots. Subsequent attempts in this field focus on improving the energy conversion efficiency to accelerate the development of molecular robots towards practical applications.

## Conclusion

The multi-functionality, programmability, and controllability of building materials for molecular robots engender molecular robots with superior potential to other types of microrobots. However, constructing molecular robots is not an easily achieved task, as it requires not only parts with high quality but also great synergy between each part. In this review, we have introduced several recent advances in the core parts of molecular robots. Looking to the future, what should be the direction of development for these core parts?

The lifetime of a GUV is reported to be up to several months,^335^ in contrast to the 10 to 20 years' average lifetime of industrial robots,^336^ reflecting a great hurdle in the successful implementation of molecular robots. Adding a second component to the lipid membranes, for example, polymers^337,338^ or surfactants,^339^ could make a giant leap in developing GUVs with higher stability. However, these impurities would also change the permeability of lipid membranes and impair the functionality of the sensors, computers, and actuators. A comprehensive list of such components with their advantages and drawbacks will have to be obtained using both experimental and *in silico* approaches. In addition, while the water/oil emulsion technology-based strategy is the leading contender for GUV fabrication, adding polymers and surfactants may change the interfacial tension between water and oil, making GUVs fabrication difficult. Consequently, new GUVs fabrication methods must be continuously explored.

For nanopores, future improvements should be directed towards increasing their homogeneity and imparting selectivity. So far, we have shown that each of the building materials for nanopores provides unique benefits: proteins are highly compatible with lipid membranes, peptides are easy to synthesize, DNA offers high programmability, and synthetic materials offers strong operability. Accordingly, combining these materials has gained great interest as a unique strategy to achieve homogeneity and selectivity. Recent reports have demonstrated methods to tune the diameter of protein^340,341^ and peptide^342,343^ nanopores using DNA nanostructures/origami as a scaffold. Such DNA nanostructures could also impart selectivity to the protein/peptide nanopores, whilst the incorporation of synthetic materials is also worth exploring.

Building a molecular computer that can perform tasks in an intelligent way like a human being is the ultimate goal for molecular robotics. The first step towards intelligence is the ability to learn. Neural networks are the backbone of deep learning algorithms, and a neural network capable of 144-bit pattern recognition with a two-layer implementation function has already been achieved using DNA computing.^205^ However, research into DNA computers with the capability to learn remains in the early phase due to the challenges in scalability (this is discussed in detail in a survey by Nagipogu *et al.*^344^). Consequently, new methods to address the scalability problems are highly desired. On the other hand, computers based on CFPS are still in their infancy. Whilst arithmetic operation in digital computers is based on a combination of logic gates, the use of protein logic gates is rarely reported. One difficult issue is the limited pool of building blocks due to the complicated interaction between proteins. Recently, Chen *et al.* reported a *de novo* design of three-input protein logic gates, giving careful consideration to the interaction between each building block.^345^ Rapid advances in *de novo* design will bring out the full potential of CFPS as computers for molecular robots, and the great diversity in the types of functional outputs of CFPS may lead to new breakthroughs in computational intelligence.

Despite the significant progress in actuating lipid membranes with DNA nanostructures/origamis and proteins/peptides, most of the reported methods rely on uncontrollable thermal fluctuation-dependent molecular processes, hence restricting their operability. Applying a controllable external field may help meet this challenge, as achievements in the actuation of DNA nanostructures/origamis with both electric^346^ and magnetic fields^347^ have progressed in recent years. The incorporation of electrochemically, magnetically, or acoustically sensitive chemical groups would allow development of increasingly superior actuators for molecular robots.

Improving the cooperation between sensors, computers, and actuators is the key to realizing the potential of molecular robots. Previous molecular robots mainly utilize open-loop control systems, meaning that the output generated by computers or actuators does not affect the input from sensors. Though the simplicity of such an open-loop control system offers many benefits, its poor accuracy limits the reliability of molecular robots. In conventional mechanical engineering, closed-loop control systems are known to improve accuracy, and we argue that the same rule-of-thumb should be valid for molecular robots. Only a limited number of molecular robots utilizing closed-loop systems have been reported. In one example, Peng *et al.* developed a molecular robot that could detect Sr^2+^ with a DNA computer while regulating the Sr^2+^ input from DNA nanopores^141^ (this is also discussed in the section on Applications of Molecular Robots). Usage of sensors, computers, and actuators that can precisely control the function of each other is expected to become mainstream in molecular robots' development.

Another challenge that needs to be addressed is how to maintain molecular robots' sustainability. The working principles of current molecular robots follow the minimization of free energy, limiting the lifetime of their functionalities. Looking ahead toward a versatile molecular robot, the ability to sustain non-equilibrium conditions where energy and materials are continuously supplied is highly desirable. As we discussed earlier, the introduction of a self-fuelling system may solve the energy supply issue (readers are referred to the section on Applications of Molecular Robots for details). On the other hand, the requirement to supply new material can be addressed by utilizing membrane fusion. Zhao *et al.* have demonstrated that CFPS in GUVs can be triggered by the fusion of GUVs and large unilamellar vesicles encapsulating DNA plasmids.^348^ Enabling sustained out-of-equilibrium operation represents a critical step towards the deployment of molecular robots for practical application.

Looking at the success of currently reported applications of molecular robots, how can these applications be further expanded in combination with other technologies? Wearable sensors, which are electronic devices that can be comfortably worn, provide promising approaches to monitor physiological information in real-time.^349^ To provide sensing selectivity and specificity, biological systems such as livening engineered bacteria^350^ and CFPS^351^ have been incorporated into wearable sensors. Compared with previous systems, molecular robots can combine both the functionality of living organisms and the durability of CFPS, making them a strong candidate for new wearable sensors. Another likely trend will be using molecular robots in the atmosphere to perform tasks such as monitoring atmospheric pollution or delivery of specific substances. While this may seem to run contrary to the principle of molecular robots, due to the absence of aqueous media, we consider the soap bubbles as a potential solution to address this issue because they are stable in the atmosphere and can trap molecules in their wall. Inspired by some interesting studies that used soap bubbles to detect methamphetamine in aerosols^352^ or to pollinate flowers,^353^ we expect that such soap bubble-based molecular robots incorporating sensors, computers, and actuators can be realized in the near future.

In conclusion, the molecular robot is the fruit of numerous technological progresses in biophysics, biochemistry, and systems engineering. With an increasing number of reports on new findings in these areas, we believe the development of molecular robots will be further accelerated, opening the door to a new era in robotics.

## Author contributions

Z. P. and R. K. conceptualized the article. Z. P. prepared the initial draft of section: Introduction, Applications of Molecular Robots, and Conclusions. Y. E. prepared the draft of section Body of Molecular Robots. Z. P., S. I., M. Y., and S. F. prepared the draft of section: Sensors of Molecular Robots. T. M., S. T., G. F, and F. K. prepared the draft of section: Computers of Molecular Robots. L. D. M., Z. P. and K. I. prepared the draft of section: Actuators of Molecular Robots. Z. P., H. S. and A. C. edited the manuscript with expert guidance, suggested edits, and supervision from R. K.

## Conflicts of interest

There are no conflicts to declare.

## Supplementary Material
